# A cross-kingdom metabolite signal: tryptophan from *Bacillus amyloliquefaciens* triggers *Ophiocordyceps sinensis* morphogenesis via the TOR-*BRG1* pathway

**DOI:** 10.3389/fmicb.2026.1818098

**Published:** 2026-06-01

**Authors:** Muhammad Zaryab Khalid, Xuehong Zheng, Richou Han, Li Cao

**Affiliations:** 1 Guangdong Key Laboratory of Animal Conservation and Resource Utilization, Guangdong Public Laboratory of Wild Animal Conservation and Utilization, Institute of Zoology, Guangdong Academy of Sciences, Guangzhou, China; 2 State Key Laboratory of Agricultural and Forestry Biosecurity, Fujian Agriculture and Forestry University, Fuzhou, China

**Keywords:** *Bacillus amyloliquefaciens*, Chinese cordyceps, cross-kingdom signaling, microbe-mediated signaling, *Ophiocordyceps sinensis*, TOR signaling

## Abstract

The development of the Chinese cordyceps relies on the efficient transformation of blastospores to hyphae of the high-value medicinal fungus *Ophiocordyceps sinensis*, which occurs in the hemolymph of *Thitarodes*. While host-associated microbiota are thought to facilitate this process in nature, the fundamental processes involved are still unclear. We identified the host associated bacterium *Bacillus amyloliquefaciens* as a potent microbial symbiont that dramatically enhances the blastospore-to-hypha transition in *O. sinensis*, increasing its transformation rate from 23.2 to 58.8% at day 8. Through a combination of bioassays, transcriptomics, and functional validation, we delineate a novel cross-kingdom signaling pathway underlying this phenomenon. Our transcriptomic analysis revealed that the *B. amyloliquefaciens* supernatant triggers extensive transcriptional reprogramming in the *O. sinensis*, upregulating the TOR signaling pathway and the key transcription factor involved in spore–hyphae transformation, including *BRG1*. Functional studies confirmed that bacterial-derived tryptophan is the key signaling metabolite modulating this TOR-*BRG1* regulatory axis. The expression of hypha-specific genes and critical cell wall remodeling enzymes (Chitin Synthase 2 and 3, Hyphal Wall Protein 1, agglutinin-like sequence 3 protein, and β-1,3-glucan synthase) were also upregulated. Our findings elucidate a previously unknown bacterial-fungal communication mechanism in which a *B. amyloliquefaciens* tryptophan directly regulates *O. sinensis* development by activating a conserved TOR-*BRG1* morphogenetic pathway. This presents a transformative biological strategy to overcome a major bottleneck in the sustainable production of Chinese cordyceps.

## Graphical Abstract

**Figure:**
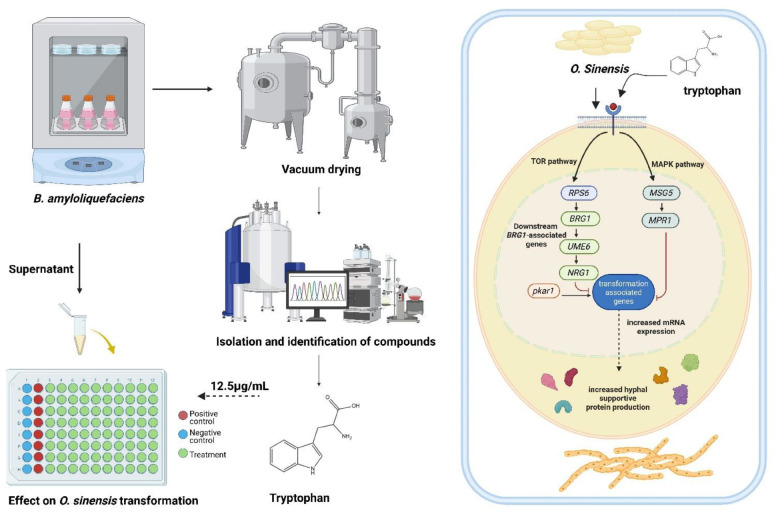
The metabolite tryptophan from *Bacillus amyloliquefaciens* modulates the fungal TOR pathway, upregulating *RPS6*. This modulates the transcription factor *BRG1*, which promotes *UME6* and represses *NRG1*, initiating hyphal development. Key cell wall remodeling enzymes (CHS2, CHS3, FKS1) are also upregulated, supporting the blastospore-to-hypha transformation.

## 1 Introduction

The fungus *Ophiocordyceps sinensis*, a well-known entomopathogen, infects and grows within the larvae of ghost moths (*Thitarodes* spp.) (Wei et al., 2021; Wu et al., 2022). This unique fungus-insect complex, known as Chinese cordyceps, ranks among the most esteemed remedies in traditional medicine in Asia due to its wide spectrum of bioactivities, including immunomodulatory, antitumor, and antioxidant properties (Han et al., 2019).

The natural production of Chinese cordyceps, however, is a slow process. The fungus can persist within the host larva for over a year as blastospores before culminating in host mummification and the formation of the characteristic fruiting body (Meng et al., 2015; Rao et al., 2019). This protracted development, combined with harsh ecological conditions and overharvesting driven by immense market demand, has led to a sharp decline in natural population (Wei et al., 2021). As a result, *O. sinensis* has been listed as an endangered species in China (Dai et al., 2020). The soaring market price, which can exceed up to $40,000- per kilogram for high-quality specimens, underscores the critical need for sustainable production methods (Lo et al., 2013). Artificial cultivation is essential to balance conservation with market demands, but it remains hindered by low infection rates and inefficient blastospore-to-hypha transformation which is a critical step for host mummification (Liu et al., 2019; Qin et al., 2018).

Emerging evidence underscores that the microbial communities inhabiting the ghost moth larva and its surrounding soil environment are pivotal in modulating the infection and subsequent development of *O. sinensis* (Liu et al., 2021; Sun et al., 2023). For example, dynamic shifts in the larval gut and hemolymph microbiota have been observed following *O. sinensis* infection, revealing a sophisticated tripartite interplay between the host insect, the pathogenic fungus, and resident microbes. High-throughput sequencing has revealed that a high load of *O. sinensis* blastospores in the hemolymph sharply reshapes the host’s internal microbial landscape, significantly increasing bacterial diversity while suppressing fungal diversity. Crucially, the larval mummification process is strongly associated with the proliferation of specific bacterial taxa, such as *Pseudomonas*, *Serratia*, and *Chryseobacterium* species, indicating that the resident microbes play a functional role in facilitating the blastospore-to-hypha transition and the subsequent successful development of Chinese cordyceps (Wu et al., 2020). *Bacillus* species are prolific producers of bioactive metabolites capable of exerting profound effects on fungal physiology and pathogenesis (Ahsan et al., 2022). These bacterial-fungal interactions span a spectrum from antagonistic to synergistic, critically influencing fungal growth and developmental transitions (Liang et al., 2019; Zhang et al., 2025).

The blastospore-to-hypha transition is a fundamental morphological shift in fungi, known to be governed by conserved signaling pathways that integrate environmental cues. These include key signaling pathways such as mitogen-activated protein kinase (MAPK), Snf1, protein kinase A (PKA), and Target of rapamycin (TOR) pathways (Sharma et al., 2019; Su et al., 2013). In the model fungus *Candida albicans*, the TOR signaling pathway is a central nutrient sensor that regulates hyphal development (Kim et al., 2024). Under conducive conditions, reduced TOR signaling triggers the upregulation of the *BRG1* transcription factor. This chromatin remodeling serves to lock in the hyphal state by occluding the binding sites for the key transcriptional repressor *NRG1*, thereby preventing the reversion to yeast growth. Furthermore, *BRG1* activates the expression of the central hyphal regulator *UME6*, establishing a powerful positive feedback loop that drives and sustains hyphal elongation (Lu et al., 2012).

Various chemical signals are known to trigger this dimorphic switch, including N-acetylglucosamine, 20-hydroxyecdysone (Liu et al., 2020), neurotransmitter acetylcholine and mannitol (Chai et al., 2024; Khalid et al., 2025). The associated bacteria in the hemolymph may be involved in the larval mummification process (Wu et al., 2021; Zeng et al., 2021). However, whether additional metabolites from these associated bacteria favor the blastospore-hypha transition in *O. sinensis* is worthy of further investigation.

In a preliminary screen of over 100 microbial isolates from the hemolymph and gut of *T. xiaojinensis* larvae, the culture supernatant of *Bacillus amyloliquefaciens* emerged as the most potent enhancer of the blastospore-to-hypha transformation in *O. sinensis*, prompting a detailed investigation into the mechanistic basis of this phenomenon. In this study, we report a novel, facilitative interaction between *B. amyloliquefaciens* and the entomopathogenic fungus *O. sinensis*. We demonstrate that bacterial-derived tryptophan acts as a key signaling metabolite that modulates an intrinsic TOR-*BRG1* regulatory axis in the *O. sinensis*, thereby driving the critical blastospore-to-hypha transition. Our findings elucidate a unique microbial cross-kingdom dialogue that governs fungal morphogenesis and present a potential biological strategy to overcome a major bottleneck in the artificial production of Chinese cordyceps.

## 2 Materials and methods

### 2.1 Strains, culture conditions, and supernatant preparation

The fungal *Ophiocordycep sinensis* (KD1223; isolated from fruiting bodies of *O. sinensis* in Sichuan, China) and the bacterial isolate *Bacillus amyloliquefaciens* (from the hemolymph of *Thitarodes xiaojinensis* larvae) were cultured and maintained in our laboratory (Khalid et al., 2025). *O. sinensis* strain KD1223 was first cultured on PPDA agar at 13 °C for 60 days. Colonies were then transferred to liquid PM medium and incubated at 13 °C with shaking at 120 rpm for 15–25 days. Briefly, blastospores were harvested by filtration through three layers of sterile lens paper, centrifuged at 8000 rpm for 15 min at 10 °C, and adjusted to a final concentration of 2 × 10^8^ blastospores per mL in phosphate-buffered saline (PBS, pH 6.2). While, *B. amyloliquefaciens* was cultured in Luria-Bertani (LB) broth at 25 °C for 4 days with shaking at 180 rpm. The culture supernatant was then collected and filter-sterilized through a 0.22 μm membrane following previous studies (Khalid et al., 2025).

### 2.2 Bioassay to evaluate the effect of host-associated *B. amyloliquefaciens* in increasing *O. sinensis* transformation

The infection process of *O. sinensis* in *T. xiaojinensis* alters the gut and hemolymph microbiota (Wu et al., 2020). To assess the potential role of screened microbiota in improving the infection rate of *O. sinensis*, a bioassay was performed in sterile 96-well microtiter plates with each well containing 90 μL of PM medium (Khalid et al., 2025). Additionally, 5 μL of filter-sterilized bacterial supernatant was added in tested wells, while control wells received 5 μL of sterile ddH_2_O. Subsequently, 5 μL of *O. sinensis* blastospore suspension (2 × 10^8^ blastospores/mL) was added to achieve a final concentration of 2 × 10^7^ blastospores/mL. Microplate shaker (Kylin-Bell, Haimen, China) was used for thorough mixing and plates were then incubated at 13°C in the dark. The percentage of blastospores transformed (%) into pre-hyphae or hyphae (combined) was recorded after 4th and 8th day of post-inoculation. Furthermore, Calcofluor White-Evans Blue stain (Sigma-Aldrich, Darmstadt, Germany) was used for staining.

### 2.3 Transcriptome profiling and differential expression analysis of *O. sinensis* following treatment with *B. amyloliquefaciens* supernatant

To investigate transcriptional responses of *O. sinensis* to *B. amyloliquefaciens* supernatant, blastospores were harvested on day 4 and day 8 post-treatment. Each condition (control and treated) included three biological replicates, resulting a total of 12 samples for transcriptome profiling. Following total RNA extraction from all samples, cDNA libraries were prepared from high-integrity RNA. Library construction involved adapter ligation and size selection with Hieff NGS^®^ DNA Selection Beads, with subsequent PCR amplification of the resulting libraries. The amplified libraries were sequenced using an Illumina NovaSeq X Plus system (Gene Denovo Biotechnology Co., Ltd, Guangzhou, China). Raw sequencing data (deposited into CNSA with accession number CNP0008141) underwent quality control processing with fastp (v0.18.0). This step eliminated adapter sequences, reads comprising more than 10% ambiguous bases (N), and reads where over 50% of bases had a quality score of 20 or lower. Bowtie2 (v2.2.8) was employed to remove ribosomal RNA sequences. The high-quality, filtered reads were then mapped to the *O. sinensis* reference genome (GenBank Assembly accession: GCA_052818365.1; BioProject: PRJNA1159368) with HISAT2 (v2.1.0). Using StringTie (v1.3.1), transcripts were assembled, and their abundance was estimated via RSEM (v1.2.19). Gene expression levels were normalized and reported as Transcripts Per Million (TPM). DESeq2 and edgeR were used to detect differential expression between groups and pairwise comparisons between samples, respectively. Differentially expressed genes (DEGs) were defined through a threshold of |log_2_ (fold change)| ≥ 1 and false discovery rate (FDR) < 0.05. Gene Ontology (GO) and Kyoto Encyclopedia of Genes and Genomes (KEGG) pathway enrichment analysis of DEGs was performed using a hypergeometric test. The calculated *p*-values were adjusted for multiple testing using the Benjamini-Hochberg FDR correction. Terms with an FDR ≤ 0.05 were considered significantly enriched.

Validation of the RNA-seq data was conducted by quantifying the mRNA expression of 12 key genes identified from the DEG and pathway analyses using quantitative real-time PCR (qRT-PCR). The qRT-PCR methodology, including reverse transcription, amplification, and specificity verification, closely followed our established protocol (Khalid et al., 2025). Several housekeeping genes, including *act*, *TEF3*, *TUB1*, and *TUBB*, were evaluated for stability. The most stably expressed gene, *TUB1*, was selected for data normalization using the 2^–ΔΔ^CT method. All primer sequences used in this study can be found in Supplementary Table S1.

### 2.4 Functional validation of the TOR-*BRG1* regulatory axis

#### 2.4.1 Transformation assays under TOR pathway modulation

To investigate the role of TOR signaling in blastospore transformation, *O. sinensis* blastospores were treated with chemical modulators of the TOR pathway, including rapamycin (CAS 53123-88-9) and farnesol (CAS 4602-84-0) purchased from Macklin Biochemical Technology Co., Ltd. (Shanghai, China). A dilution series covering 0.05 to 100 μg/mL was assessed. Additionally, to determine if the transformative effect of *B. amyloliquefaciens* supernatant was mediated through TOR signaling, co-treatment experiments combining the bacterial supernatant with rapamycin were conducted. Transformation efficiency was quantified after 4 and 8 days.

#### 2.4.2 Gene expression analysis of TOR-*BRG1* network components

The molecular mechanism underlying the phenotypic changes was investigated by quantifying the expression of key components of the TOR-*BRG1* regulatory network via qRT-PCR. Based on the transformation assay results, two concentrations of rapamycin were chosen: an inhibitory concentration (50 μg/mL) and an agonistic concentration (0.5 μg/mL). Briefly, blastospores and mycelia from both the treatment and control groups were collected by centrifugation at 8,000 rpm for 10 min. The resulting cell pellets were swiftly submerged in liquid nitrogen for flash-freezing and subsequently pulverized into a fine powder using a mortar and pestle. Total RNA was then extracted from approximately 100 mg of powdered mycelium using the HiPure Universal RNA Mini Kit (Magen Biotechnology). cDNA was synthesized from the total RNA, and qRT-PCR was carried out using the conditions as described in section 2.3. Gene expression levels were normalized to the *TUB1* and quantified via the 2^–ΔΔ^CT method.

### 2.5 Treatment of *O. sinensis* with *B. amyloliquefaciens* crude extract

To evaluate the role of metabolites, present in the supernatant, a bioassay was performed with the crude extract of *B. amyloliquefaciens* supernant. A total of 10 L of *B. amyloliquefaciens* was cultured for 4 days (optimal for metabolite production). The culture broth was filtered and then lyophilized using a vacuum freeze-dryer (Model No. TF-SFD-0.5E; Shanghai Tianfeng Industrial Co., Ltd.), yielding approximately 88.2 g of dried powder, while culture broth alone was processed in parallel as a control. This material was subjected to extraction with 2 L of 95% ethanol for three times, with each extraction lasting 24 h. The pooled ethanolic solution was filtered through a Büchner funnel and concentrated under reduced pressure at 40°C using a rotary evaporator. The resulting crude ethanol extract was further dried on a water bath. The dried extract (45.1 g) was subsequently partitioned into ethyl acetate and butanol fractions. This partition separated metabolites based on polarity, with the butanol fraction containing more polar compounds, such as amino acids and glycosides. Later the organic phases were concentrated, filtered through a 0.22 μm membrane, and dried under sterile conditions in a laminar flow cabinet. This process yielded 3.7 g of ethyl acetate-soluble fraction and 16.7 g of butanol-soluble fraction.

The bioassay was conducted in a 96-well microplate. The effect on *O. sinensis* transformation was evaluated at day 4 and day 8 post-treatment, as transcriptomic analysis showed peak gene activation at day 4 and maximum transformation efficiency at day 8. Preliminary experiments evaluating the impact of DMSO concentrations (0.1, 0.5, 1, 5, and 10%) on blastospore transformation revealed no significant difference between 0.5% DMSO and the untreated control. Therefore, the crude extracts were dissolved in 0.5% DMSO and tested at concentrations ranging from 500 to 3.9 μg/mL. Each well contained 85 μL of PM medium and 5 μL of blastospore suspension (2 × 10^8^ blastospores/mL). While, 10 μL of ethyl acetate or butanol crude extracts were added to make final volume of 100 μL. The percentage of blastospore transformation was recorded after 4 and 8 days of incubation.

### 2.6 Isolation and identification of bioactive compounds from *B. amyloliquefaciens*

To deeply investigate the role of individual compounds responsible for increased transformation observed with the butanol crude extract, this fraction was further selected for isolation and identification of compounds. Initial separation was achieved via silica gel column chromatography. A stepwise elution gradient was applied, progressing through petroleum ether/ethyl acetate (50:1–1:2, v/v), then dichloromethane/methanol (50:1–1:2, v/v), and finally pure methanol. Collected fractions were then concentrated and grouped based on their thin layer chromatography (TLC) profiles. Further purification of active sub-fractions was achieved using preparative high-performance liquid chromatography (HPLC) (Agilent technologies G1316A) equipped with an InfinityLab Poroshell 120 EC-C18 column (2.7 μm, 4.6 × 100 mm). The final purification step to obtain pure compounds was performed using an Elite P3500 HPLC system with a SinoChrom ODS-BP column (10 μm, 10.0 × 250 mm). Compounds exhibiting > 90% purity were characterized by nuclear magnetic resonance (NMR) spectroscopy. One-dimensional (^1^*H* and ^13^*C*) and two-dimensional (COSY, HMBC, HSQC) NMR experiments were employed for structural elucidation.

### 2.7 Treatment of *O. sinensis* with identified compounds

The transformative activity of the identified compounds was assessed using the bioassay detailed in section 2.5. In brief, 10 μL of each pure compound was used in place of the crude extract in a 96-well plate assay. L-phenylalanine (32-38-1; CAS 63-91-2), tryptophan (32-38-2; CAS 54-12-6), Cyclo(L-Pro-L-Val) (35-38-1; CAS 2854-40-2) and Cyclo(L-Leu-L-Pro) (35-38-3; CAS 2873-36-1) were purchased as ultra-pure grade powders from Shanghai Macklin Biochemical Technology Co., Ltd. (Shanghai, China). The transformation (%) was measured and recorded on the 4th and 8th days following post-inoculation.

### 2.8 Quantification of tryptophan from *B. amyloliquefaciens* and *O. sinensis* supernatant

To further determine the source and concentration of tryptophan involved in the observed fungal morphogenesis, the extracellular tryptophan levels in the culture supernatants of both *B. amyloliquefaciens* (after 4 days) and *O. sinensis* (after 25 days) were quantified using High-Performance Liquid Chromatography (HPLC). The supernatant of both *B. amyloliquefaciens* and *O. sinensis* was prepared as described in section 2.1.

Separation and quantification of tryptophan were achieved using an HPLC system (Agilent 1290 Infinity II) equipped with a DAD detector. The analysis was performed on an Agilent ZORBAX SB-C18 column (4.6 mm × 150 mm, 5 μm) maintained at a constant temperature of 35 °C. The mobile phase consisted of 20 mM potassium dihydrogen phosphate (KH_2_PO_4_) in ultrapure water, adjusted to pH 2.5 with phosphoric acid (Mobile Phase A), and acetonitrile (HPLC grade, Mobile Phase B). An isocratic elution was employed with a ratio of 90% Mobile Phase A to 10% Mobile Phase B for a total run time of 8 min at a constant flow rate of 1.0 mL/min. The injection volume was 3 μL, and tryptophan was detected at a wavelength of 278 nm.

### 2.9 Functional validation of tryptophan-mediated TOR-*BRG1* activation

To test the hypothesis that tryptophan acts as the bacterial metabolite activating the TOR-*BRG1* axis, the expression of key genes (*RPS6*, *BRG1*, *NRG1*, *UME6*, and *pkar1*) was evaluated after 4 days of tryptophan treatment (12.5 μg/mL) of *O. sinensis*. Total RNA was extracted from *O. sinensis* harvested after 4 days of treatment. Subsequently cDNA was synthesized and qRT-PCR was performed following protocol as described above (section 2.3).

### 2.10 Quantification of hyphal cell wall biosynthesis enzymes by enzyme-linked immunosorbent assay (ELISA)

To further support if tryptophan increase transformation by enhancing the synthesis of hyphal cell wall components, we quantified the concentration of key biosynthetic enzymes. We focused on cell wall remodeling enzymes because the blastospore-to-hypha transition requires extensive restructuring of the cell wall, a process critical for invasive growth. Specifically, the concentrations of CHS2 and CHS3 (Chitin Synthase 2 and 3), HWP1 (Hyphal Wall Protein 1), ALS3 (agglutinin-like sequence 3 protein), and FKS1 (β-1,3-glucan synthase) were quantified from *O. sinensis* following tryptophan treatment by using ELISA kits (Jiangsu Meimian Industrial Co., Ltd., Jiangsu, China) according to manufacture protocol. Briefly, samples and standards were added to the plate and incubated for 30 min at 37°C. Following washing, enzyme-specific HRP-conjugates were added and incubated for another 30 min at 37°C. The plate was then washed, after which chromogen substrates were added and allowed to incubate for 10 min at 37°C. The reaction was then stopped by adding stop solutions, and the absorbance of each well was measured at 450 nm using a Multiskan FC microplate photometer (Thermo Fisher Scientific). All measurements were performed with three biological replicates per condition. The data was recorded after 4 days of treatment.

### 2.11 Statistical analysis

Data are reported as mean ± SD and were analyzed using GraphPad Prism (version 9.0). To determine significance between two groups, we employed an unpaired two-tailed Student’s *t*-test. Comparisons across three or more groups were conducted using one-way analysis of variance (ANOVA). The figure legends detail the specific statistical test applied in each case.

## 3 Results

### 3.1 *B. amyloliquefaciens* supernatant enhances *O. sinensis* blastospores transformation

**FIGURE 1:**
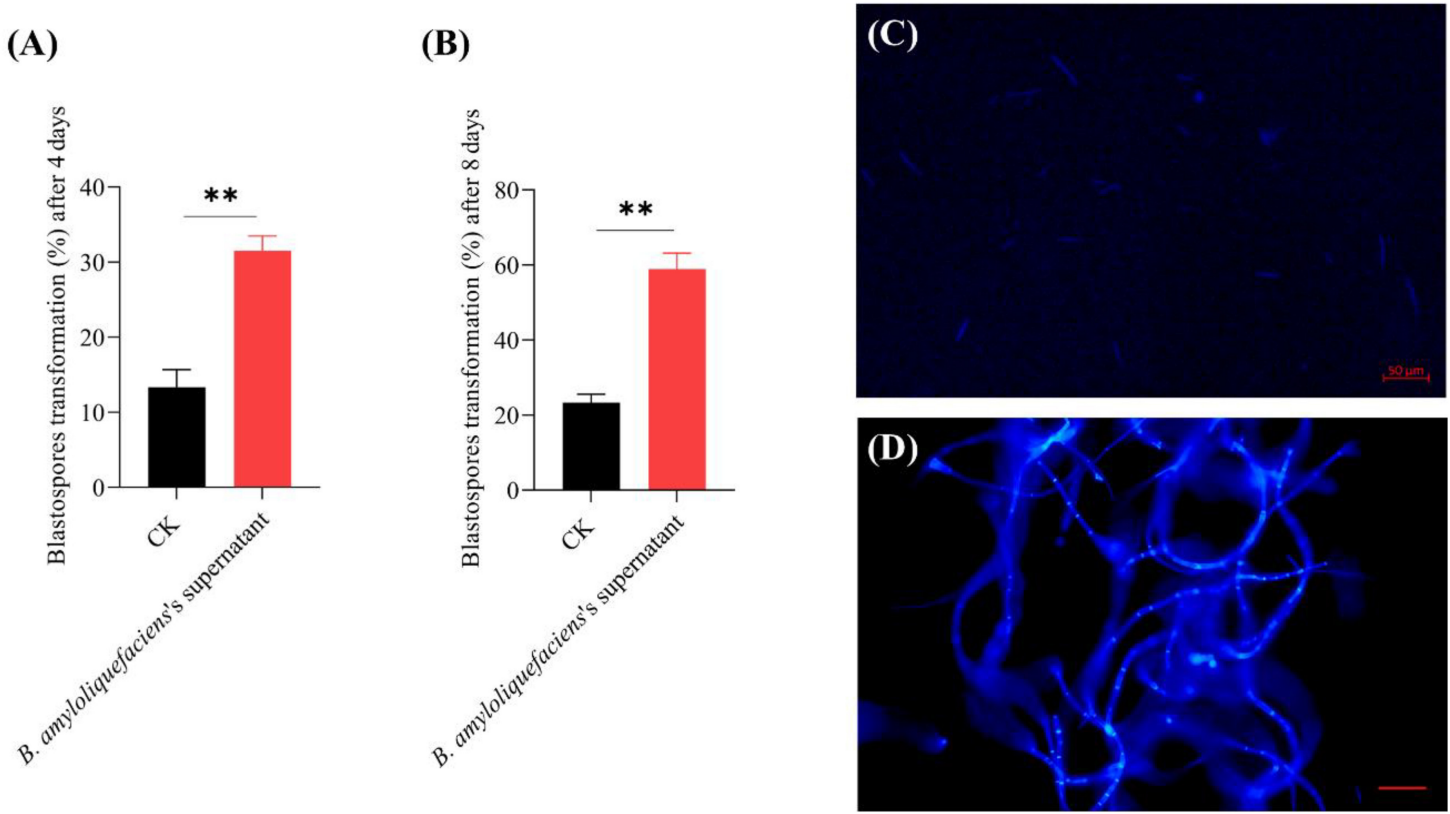
Effect of *Bacillus amyloliquefaciens* supernatant on *Ophiocordyceps sinensis* spore-to-hyphae transformation. **(A,B)** Transformation percentage at day 4 and day 8 in control (CK) and treatment groups. **(C,D)** Confocal microscopic images of the control (CK) and treatment group at day 8, respectively. Scale bar = 50 μm. Results are shown as mean ± SD. An unpaired two-tailed Student’s *t*-test was used for analysis (**p < 0.01, ns = not significant).

After 4 days of incubation, blastospores exposed to *B. amyloliquefaciens* supernatant exhibited a transformation rate of 31.5%, indicating a marked increase in transformation relative to the control (13.3%) (Figure 1A). By day 8, transformation further increased to 58.8% in the treatment group, compared to 23.2% in the control group, reflecting a substantial increase in spore-to-hyphae transition upon exposure to the bacterial supernatant (Figure 1B). Confocal microscopy confirmed these morphological changes, revealing extensive hyphal development in treated samples compared to the control (Figures 1C,D).

### 3.2 Transcriptional reprogramming of *O. sinensis* in response to *B. amyloliquefaciens* supernatant

Illumina RNA sequencing was performed on 12 libraries, which included control groups (BCK4 and BCK8) and treatment groups (BT4 and BT8). The sequencing yielded high-quality, filtered reads, with > 96% average alignment to the *O. sinensis* genome. Gene expression levels were quantified using TPM, and DEGs among treatment and control groups were identified. Functional annotation and enrichment analyses assigned DEGs to Gene Ontology (GO) categories and KEGG (Kyoto Encyclopedia of Genes and Genomes) pathways, highlighting several key biological processes and metabolic/signaling pathways responsive to the bacterial supernatant.

**FIGURE 2:**
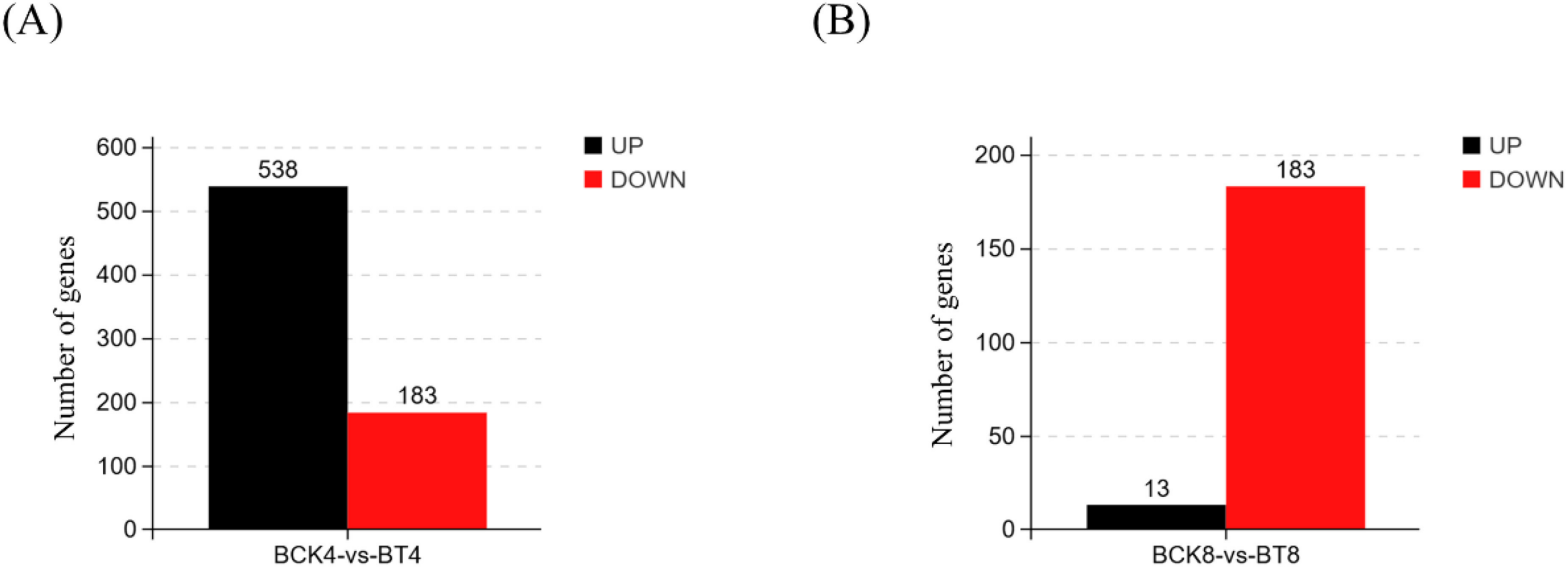
Number of differentially expressed genes (DEGs) in *Ophiocordyceps sinensis* following treatment with *Bacillus amyloliquefaciens* supernatant. **(A)** Number of DEGs at day 4. **(B)** Number of DEGs at day 8.

Transcriptomic profiling revealed a highly dynamic host response over time. At day 4, analysis revealed 721 DEGs in total (538 upregulated, 183 downregulated; Figure 2A). This number declined significantly by day 8, with only 13 upregulated and 183 downregulated genes, which reflects a major temporal shift in the transcriptional profile (Figure 2B). This temporal decline in DEG numbers reflects distinct biological phases: day 4 represents an early activation stage with widespread transcriptional reprogramming to initiate morphogenesis, whereas by day 8, the majority of blastospores have already transitioned to hyphae, and the fungus has entered a transcriptional maintenance phase with fewer active changes. Thus, the maximal increase in transformation observed at day 8 (58.8%) results from the initial transcriptional response activated at day 4.

**FIGURE 3:**
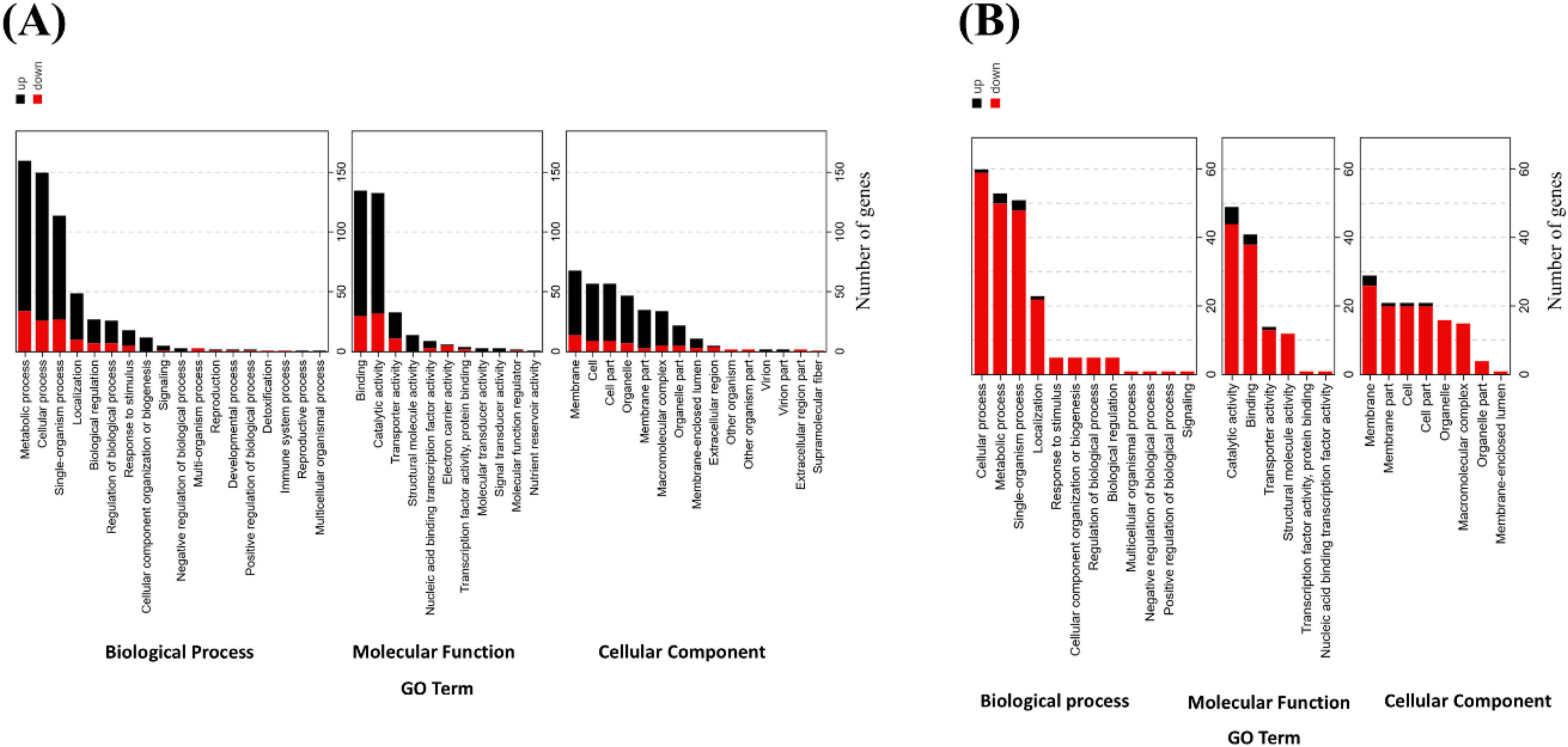
Gene ontology (GO) terms significantly enriched among the *Ophiocordyceps sinensis* differentially expressed genes (DEGs) in response to *Bacillus amyloliquefaciens* supernatant. **(A)** Enriched GO terms associated with DEGs at day 4. **(B)** Enriched GO terms at day 8. Only significantly enriched terms (FDR ≤ 0.05) are shown.

GO enrichment analysis indicated pronounced transcriptional activation in *O. sinensis* after 4 days of treatment with *B. amyloliquefaciens* supernatant (Figure 3A). Upregulated genes were mainly associated with biological processes such as “metabolic process” and “cellular process,” reflecting enhanced signaling and metabolic activity required for initiating morphological changes. Enriched molecular functions, including “catalytic activity” and “binding,” point to increased enzymatic functions supporting biosynthesis and cellular remodeling. In the cellular component category, terms such as “cell part” and “rganelle” were enriched, indicating that these DEGs are associated with internal cellular structures. Overall, these results highlight an early transcriptional reprogramming phase that likely contributes to the initial increase in spore-to-hyphae transformation. By day 8, transcriptional activity had declined (Figure 3B), with fewer genes upregulated and the majority downregulated. Although some DEGs remained linked to “developmental processes,” this reduced enrichment suggests that *O. sinensis* had progressed from the initial activation phase to a stabilization stage, where hyphal development continues under maintained transcriptional programs (Figure 3B).

**FIGURE 4:**
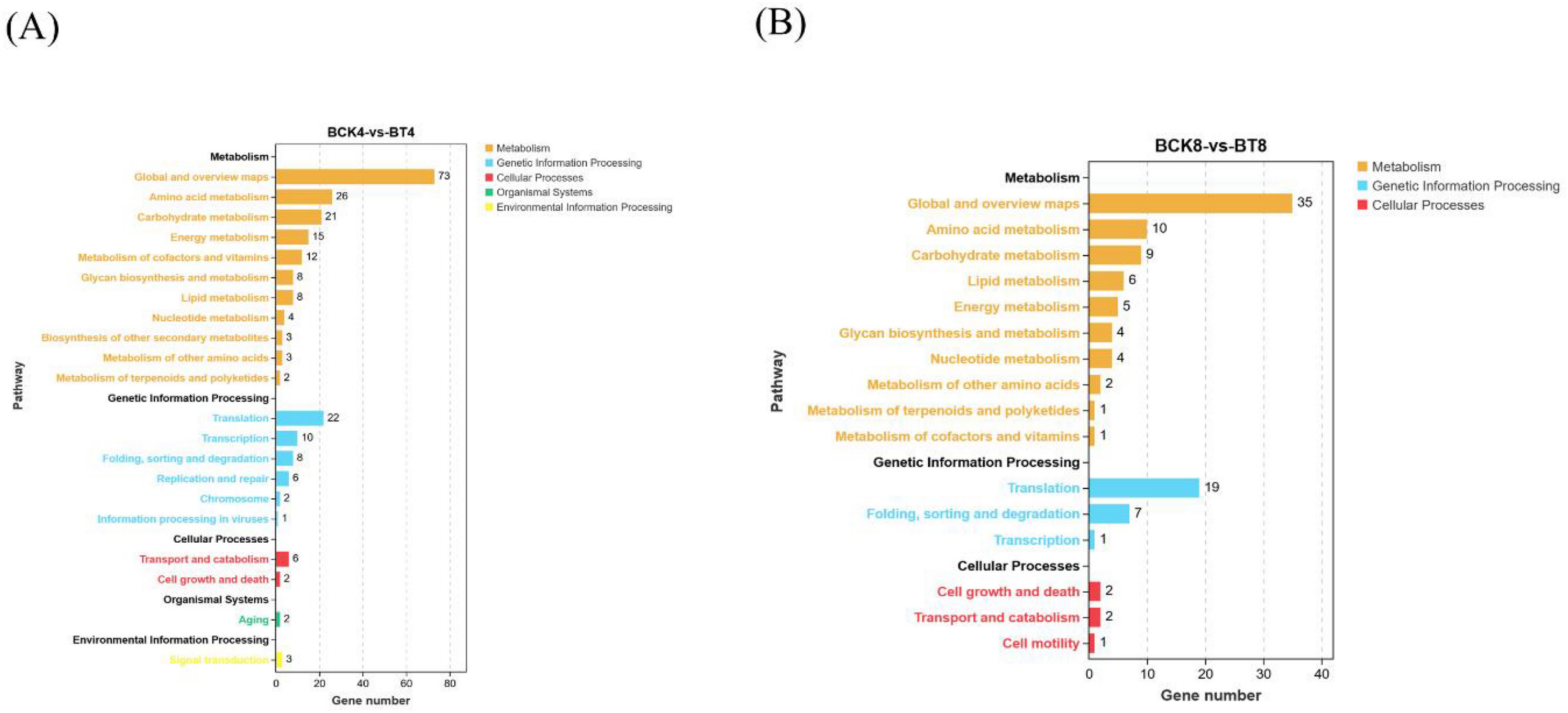
KEGG pathways significantly enriched for *Ophiocordyceps sinensis* differentially expressed genes (DEGs) after treatment with *Bacillus amyloliquefaciens* supernatant. **(A)** Enriched KEGG pathways associated with DEGs at day 4. **(B)** Enriched KEGG pathways at day 8. Pathways are categorized into metabolism, genetic information processing, and cellular processes. Only significantly enriched terms (FDR ≤ 0.05) are shown.

Additionally, KEGG pathway enrichment analysis revealed marked transcriptional activation on day 4. Upregulated genes were mainly enriched in metabolism-related pathways, including “amino acid metabolism,” “carbohydrate metabolism,” and “energy metabolism,” consistent with the elevated biosynthetic and energy requirements of spore-to-hyphae transition. Additional enrichment in genetic information processing pathways such as “translation,” and “transcription” reflected increased protein synthesis and turnover essential for cellular remodeling and development (Figure 4A). By day 8, the number of enriched pathways and DEGs decreased substantially. While some genetic information processing pathways remained active, most metabolic and cellular processes showed reduced activity, aligning with the GO results and indicating a transition from transcriptional activation to maintenance of the hyphal state (Figure 4B).

Briefly, pathways related to amino acid metabolism and central carbon metabolism were significantly upregulated at day 4, indicating a global increase in metabolic activity and biosynthetic capacity to support the energy and building blocks required for hyphal morphogenesis. Expression analysis identified a significant upregulation of the transcription factor gene *BRG1* on day 4 post-treatment, a GATA-type transcription factor associated with hyphal development. This timing coincides with the critical window for spore-to-hypha transformation, implicating *BRG1* in the initiation of hyphal development. Functionally, *BRG1* is associated with the GO terms sequence-specific DNA binding (GO:0043565), zinc ion binding (GO:0008270), and regulation of transcription, DNA-templated (GO:0006355), consistent with its role as a DNA-binding transcriptional regulator. This leads to the activation of hypha-associated genes by facilitating chromatin remodeling, primarily through the recruitment of histone deacetylases like Hda1. The significant induction of *BRG1* suggests it acts as a key regulatory switch, activating the genetic program necessary for spore-to-hypha transformation. To explore how this *BRG1*-driven morphological program is integrated with broader cellular responses, we analyzed the expression of genes associated with major signaling pathways. The Target-of-Rapamycin (TOR) pathway, a master regulator that coordinates nutrient sensing with growth and ribosomal biogenesis (Kim et al., 2024), was of particular interest. Our data show that the transcript levels of *RPS6*, a ribosomal protein and a direct downstream component of the TOR pathway (Lu et al., 2012), was significantly upregulated. This coordinated upregulation of a key transcriptional regulator (*BRG1*) and a core growth-related effector (*RPS6*) point to a synchronized activation of developmental and anabolic processes essential for hyphal formation. Alongside the central TOR-*BRG1* axis, transcriptomic analysis indicated a notable enrichment of the MAPK signaling pathway (NES = 1.37, nominal p* < 0.05), a key regulator of fungal development and stress responses. Although this enrichment did not meet the stringent FDR threshold (FDR = 0.17), the pathway’s upregulation is supported by the increased expression of core constituent genes, including *MSG5* and *MPR1* at day 4. Given the well-established role of MAPK signaling in controlling hyphal morphogenesis, these collective findings suggest its potential contributory role in the transformation process and highlight it as a pathway of interest for future mechanistic validation.

To confirm the reliability of the RNA-seq results, the mRNA levels of key candidate genes were assessed using qRT-PCR. After 4 days, genes such as *IDH1*, *ilv-2*, *Cdase*, *eca39* and *stt3* were upregulated (1. 76-, 1. 68-, 6. 1-, 2. 25-, and 2.78-fold, respectively), while *Dbt* showed no significant change (Supplementary Figure S1A). By 8 days, *PaAT-1* and *BEA3* were upregulated (4.56- and 2.32-fold), whereas *CSY2*, *ilv-2*, *ARG1* and *IDH1* were downregulated (0. 33-, 0. 71-, 0. 59-, and 0.63-fold, respectively) (Supplementary Figure S1B). Overall, the qRT-PCR analysis verified the gene expression patterns observed in the RNA-seq dataset. The consistent transcriptomic and qPCR data confirm that the bacterial supernatant triggers a major shift in fungal metabolism and gene expression, priming the cells for growth. These transcriptomic results indicated synchrony between a developmental transcription factor and a translational effector, which might suggest that the TOR pathway acts as an upstream master switch.

### 3.3 The TOR Pathway directs spore-to-hypha transformation via the *BRG1* transcriptional network

#### 3.3.1 Rapamycin exerts a concentration-dependent, biphasic effect on transformation

**FIGURE 5:**
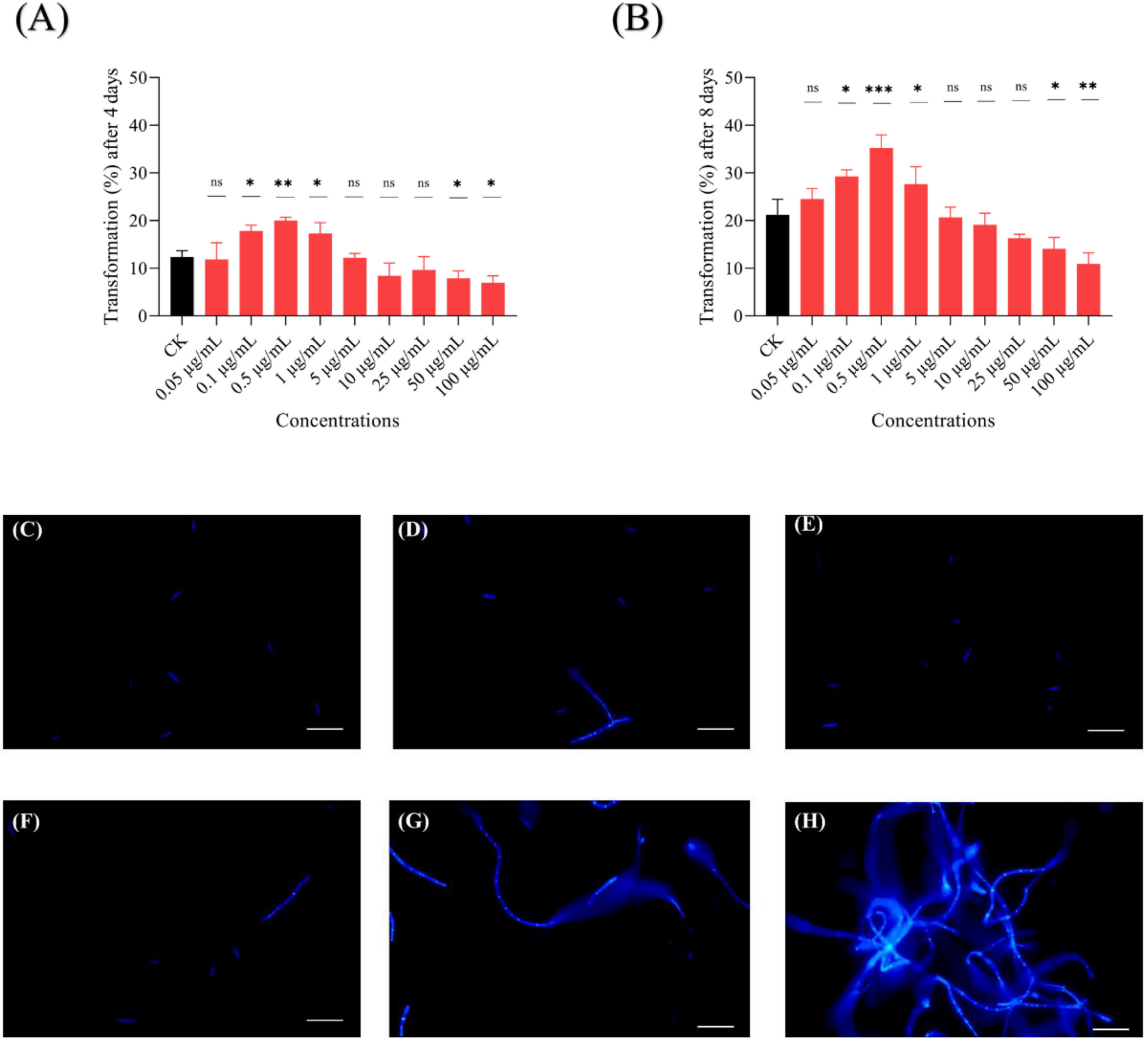
Effect of TOR signaling modulation by rapamycin on *Ophiocordyceps sinensis* spore-to-hypha transformation. **(A,B)** Transformation rates following rapamycin treatment at day 4 and day 8, respectively. **(C,D)** Confocal microscopic images of blastospores from control group (CK) at day 4 and day 8. **(E,F)** Confocal microscopic images of blastospores treated with higher concentrations of rapamycin (50 μg/mL) at day 4 and day 8. **(G,H)** Images of spores treated with lower concentrations of rapamycin (0.5 μg/mL) at day 4 and day 8. Scale bar = 50 μm. Data are presented as mean ± SD and analyzed using one-way ANOVA followed by Dunnett’s *post-hoc* test (*p < 0.05, **p < 0.01, ***p < 0.001, ns, not significant).

To directly test the role of TOR signaling in the developmental switch, we treated *O. sinensis* spores with the different TOR modulators, including rapamycin and farnesol. We observed that rapamycin exerted a concentration-dependent, biphasic effect on the spore-to-hypha transformation (Figure 5). At low concentrations (0.1–1 μg/mL) the transformation was significantly increased. The transformation rate increased from 12.4% in the control to a peak of 20.0% at 0.5 μg/mL, at day 4. In contrast, higher concentrations reduced the transformation rate to 7.9% at 50 μg/mL (Figure 5A).

Our results further indicated that this biphasic pattern persisted after 8 days. The transformation rate in the control group reached 21.2%, which was further increased to 35.2% with 0.5 μg/mL of rapamycin treatment. Conversely, the 50 μg/mL treatment significantly suppressed the transformation rate to 14.1%, compared to the control group (Figure 5B). Confocal microscopy confirmed these concentration-dependent morphological differences, with extensive hyphal networks at low rapamycin concentrations versus limited transformation at high concentrations (Figures 5C–H). Moreover, the transformation efficiency was significantly suppressed in a concentration-dependent manner by farnesol treatment (Supplementary Figure S2).

#### 3.3.2 TOR signaling regulates *BRG1* network gene expression

To deeply investigate the molecular basis of the biphasic effect of rapamycin on *O. sinensis* transformation, we quantified the mRNA levels of genes associated with the TOR–*BRG1* regulatory axis after treatment with low (0.5 μg/mL) and high (50 μg/mL) concentrations of rapamycin.

**FIGURE 6:**
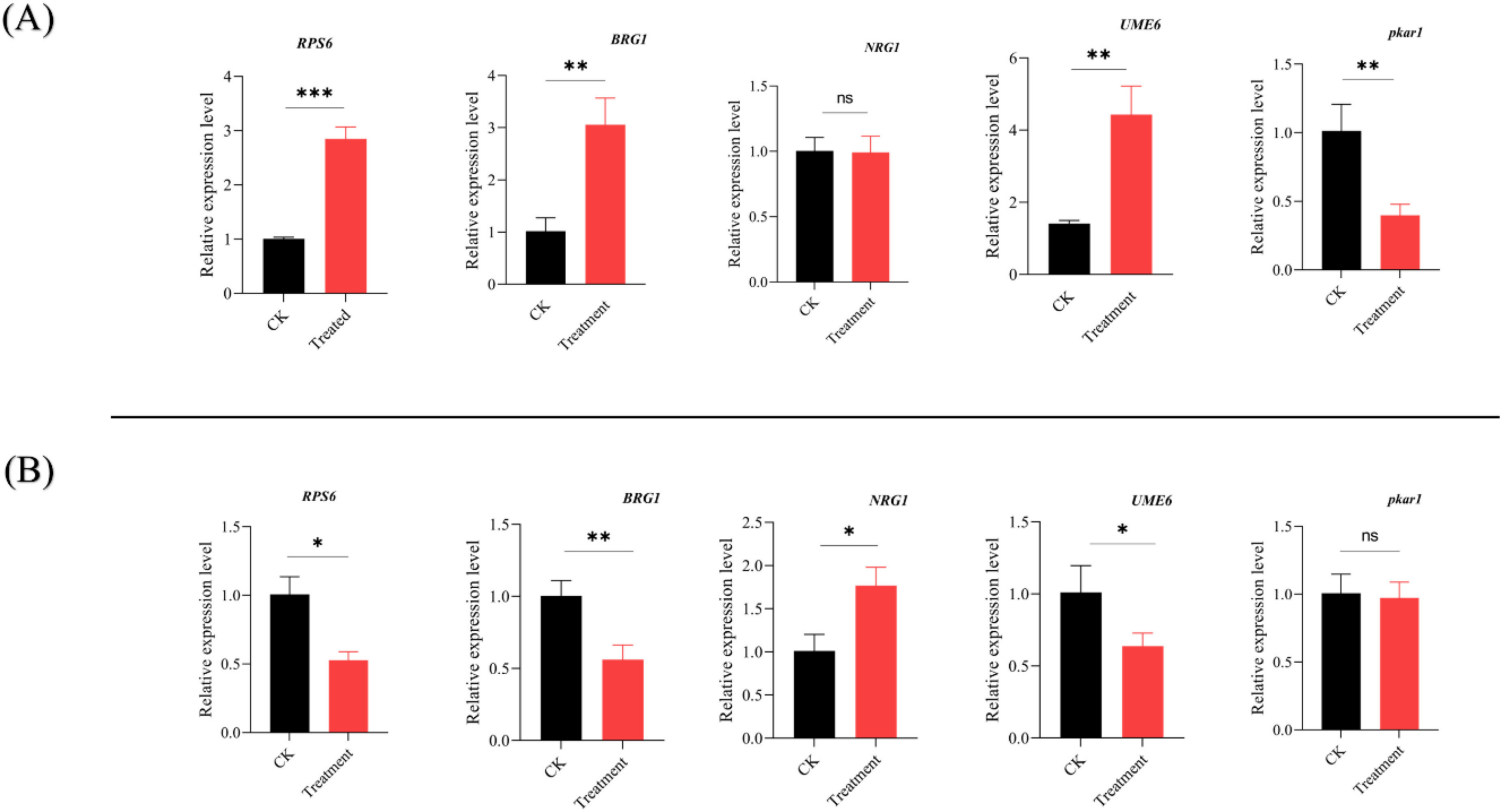
Transcriptional response of the TOR–*BRG1* regulatory network to rapamycin treatment in *Ophiocordyceps sinensis*. **(A)** Relative mRNA expression levels of *RPS6*, *BRG1*, *UME6*, *NRG1*, and *pkar1* after treatment with a low concentration of rapamycin (0.5 μg/mL) at day 4. **(B)** Relative mRNA expression levels of *RPS6*, *BRG1*, *UME6*, *NRG1*, and *pkar1* following treatment with high concentration of rapamycin (50 μg/mL) at day 4. Results are shown as mean ± SD. An unpaired two-tailed Student’s *t*-test was used for analysis (*p < 0.05, **p < 0.01, ***p < 0.001, ns, not significant).

At the low concentration, rapamycin modulated TOR signaling to a permissive level, significantly up-regulating *RPS6* at day 4. Interestingly, the transcription factor *BRG1* and its downstream developmental regulator *UME6* were markedly induced, whereas *NRG1* expression remained unchanged. In contrast, the cAMP-PKA regulatory subunit *pkar1* was significantly down-regulated, indicating a potential shift in signaling favoring hyphal differentiation (Figure 6A).

Conversely, a high concentration of rapamycin (50 μg/mL) produced the opposite pattern: transcript levels of *RPS6*, *BRG1*, and *UME6* declined sharply, while *NRG1* expression increased. The inverse relationship between *BRG1*/*UME6* and *NRG1* expression suggests that excessive TOR inhibition represses the *BRG1*-dependent developmental network, possibly through *NRG1*-mediated negative regulation. Together, these results demonstrate that TOR signaling exerts a concentration-dependent control over the *BRG1* transcriptional network: a precisely modulated level of TOR signaling enhances hyphal-specific gene expression and transformation, whereas excessive inhibition suppresses this pathway by stimulating *NRG1* expression (Figure 6B). However, farnesol treatment did not affect the mRNA expression of *BRG1* and its associated genes (Supplementary Figure S3).

### 3.4 Bioactivity of crude extracts from *B. amyloliquefaciens*

**FIGURE 7:**
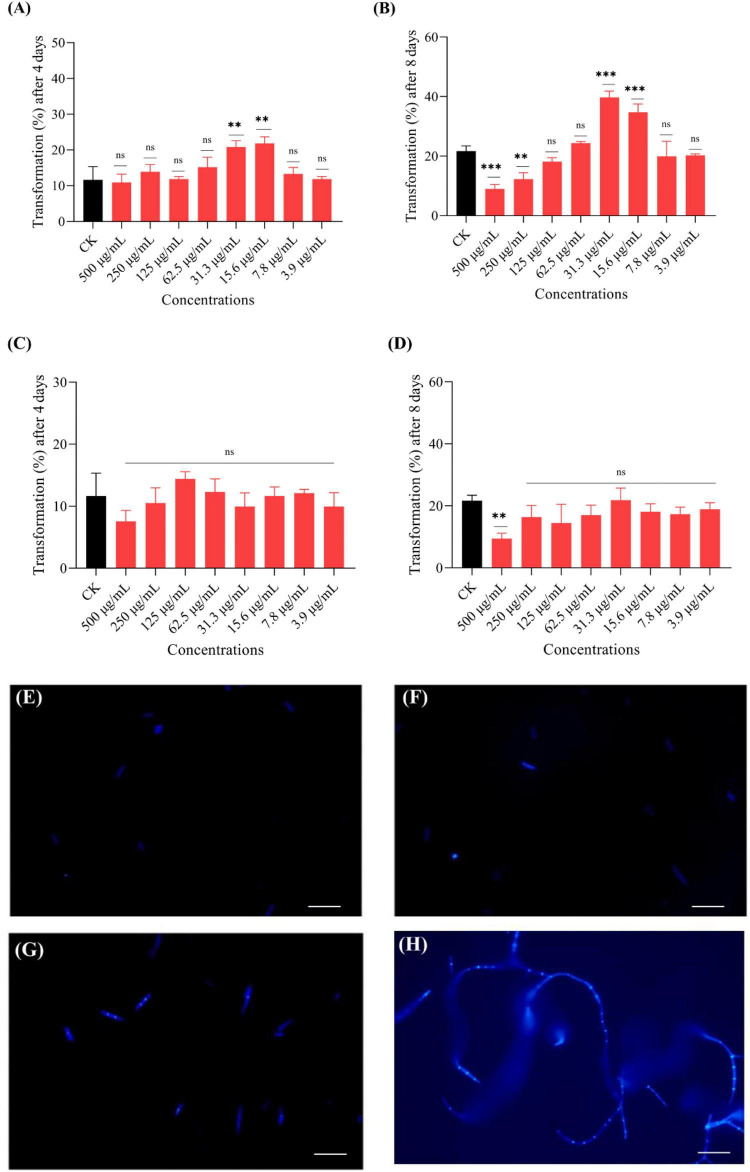
Effect of *Bacillus amyloliquefaciens* extracts on *Ophiocordyceps sinensis* blastospore-to-hyphae transformation. **(A,B)** Transformation (%) after treatment with butanol extract at different concentrations on day 4 (**A**) and day 8 **(B)**. **(C,D)** Transformation after treatment with ethyl acetate extract on day 4 **(C)** and day 8 **(D)**. **(E,H)** Confocal microscopic images: **(E)** control at day 8, **(F)** ethyl acetate-treated (31.3 μg/mL) at day 8, **(G)** butanol extract-treated (31.3 μg/mL) at day 4, and **(H)** butanol extract-treated (31.3 μg/mL) at day 8. Data are presented as mean ± SD and analyzed using one-way ANOVA followed by Dunnett’s *post-hoc* test (**p < 0.01, ***p < 0.001, ns, not significant).

Our results indicated that the butanol extract significantly promoted transformation in a concentration-dependent manner. On day 4, transformation increased from 11.6% in the control to 21.9% at 15.6 μg/mL and 20.8% at 31.3 μg/mL (Figure 7A). On day 8, 15.8 μg/mL and 31.3 μg/mL of butanol treatment enhanced transformation to 34.7 and 39.7%, respectively, compared to 21.7% in the control (Figure 7B). Higher concentrations (250–500 μg/mL) inhibited transformation. These results indicate that the butanol extract contains metabolites that actively regulate blastospore-to-hyphae transformation in *O. sinensis*. In contrast, ethyl acetate extract had no significant effect on *O. sinensis* transformation and caused a decrease at 500 μg/mL on day 8 (Figures 7C,D). Confocal microscopy visually confirmed these effects, showing robust hyphal development in butanol-treated cultures compared to limited morphological changes in controls and ethyl acetate-treated groups (Figures 7E–H).

### 3.5 Structure elucidation of the identified compounds

**FIGURE 8:**
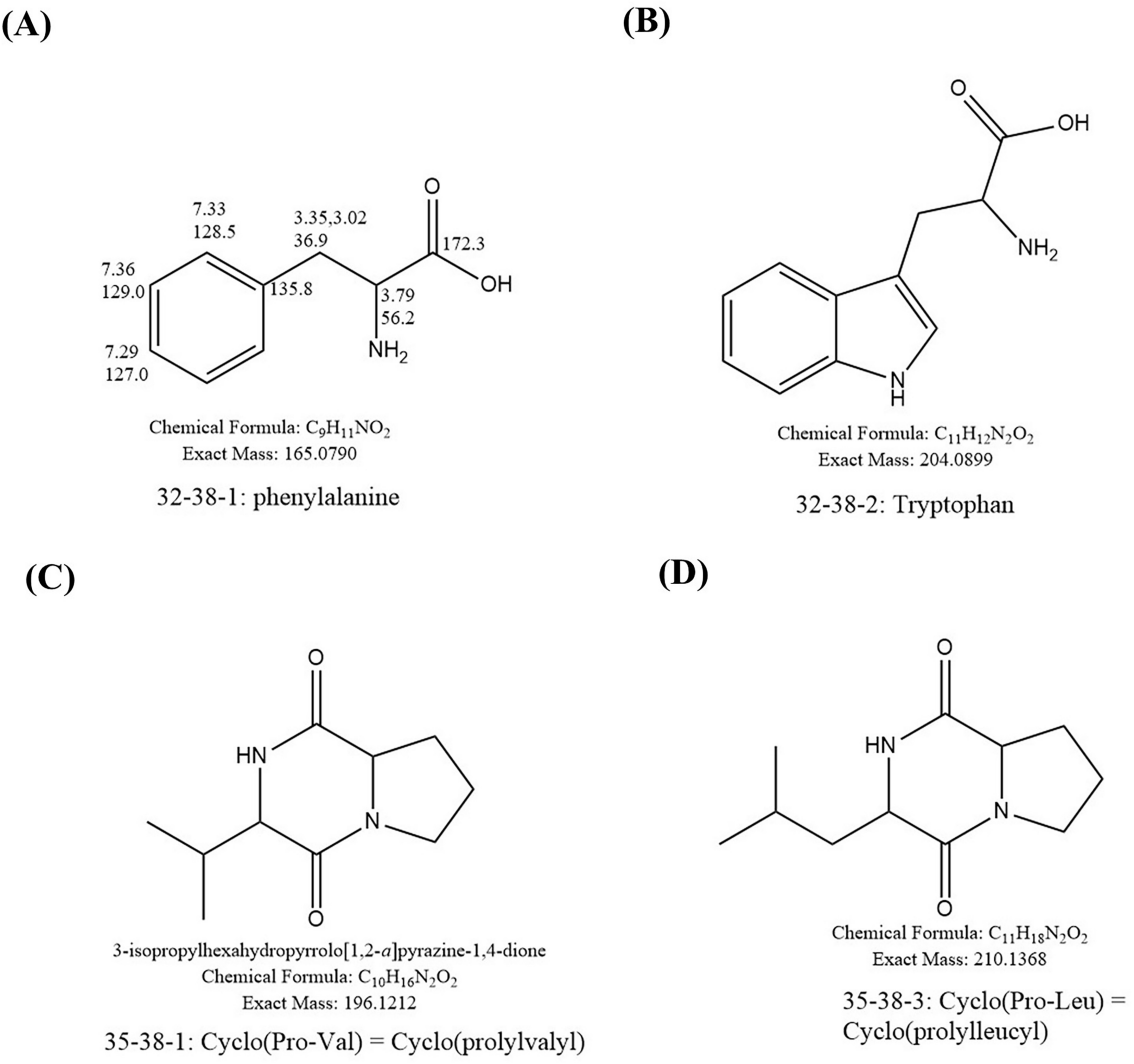
Pure compounds isolated from the butanol crude extract of *Bacillus amyloliquefaciens*. Four metabolites were identified: **(A)** L-Phenylalanine, **(B)** Tryptophan, **(C)** Cyclo(L-Pro-L-Val), and **(D)** Cyclo(L-Pro-L-Leu).

Using the butanol fraction of the *B. amyloliquefaciens*, four pure compounds—L-phenylalanine, tryptophan, cyclo(L-Pro-L-Val), and cyclo(L-Leu-L-Pro)—were isolated. Their structures were unequivocally identified (Figure 8) by mass spectrometry to confirm molecular mass and by a combination of 1D NMR (^1^H and DEPT) and 2D NMR (COSY, HSQC, and HMBC) experiments for full structural elucidation (Supplementary Figure S4).

Compound 1: L-phenylalanine; white crystal; molecular formula: C_9_H_11_NO_2_. The identity of Compound 1 was confirmed by comparison with literature (Chou et al., 2021) (NMR data shown in Supplementary Figure S4A).

Compound 2: Tryptophan; white crystal; molecular formula: C_11_H_12_N_2_O_2_. The identity of Compound 2 was confirmed by comparison with literature (Qu et al., 2022) (NMR data shown in Supplementary Figure S4B).

Compound 3: Cyclo(L-Pro-L-Val); white crystal; molecular formula: C_10_H_16_N_2_O_2_. The identity of Compound 3 was confirmed by comparison with literature (Lee et al., 2021) (NMR data shown in Supplementary Figure S4C).

Compound 4: Cyclo(L-Leu-L-Pro); white crystal; molecular formula: C_11_H_18_N_2_O_2_. The identity of Compound 4 was confirmed by comparison with literature (Yoshinari et al., 2007) (NMR data shown in Supplementary Figure S4D).

The structural formula, mass spectrum and structural presentation of the compounds are shown in Figure 8. These compounds were further evaluated for their activity in promoting blastospore-to-hyphae transformation of *O. sinensis*.

### 3.6 Effect of isolated compounds on transformation of *O. sinensis*

**FIGURE 9:**
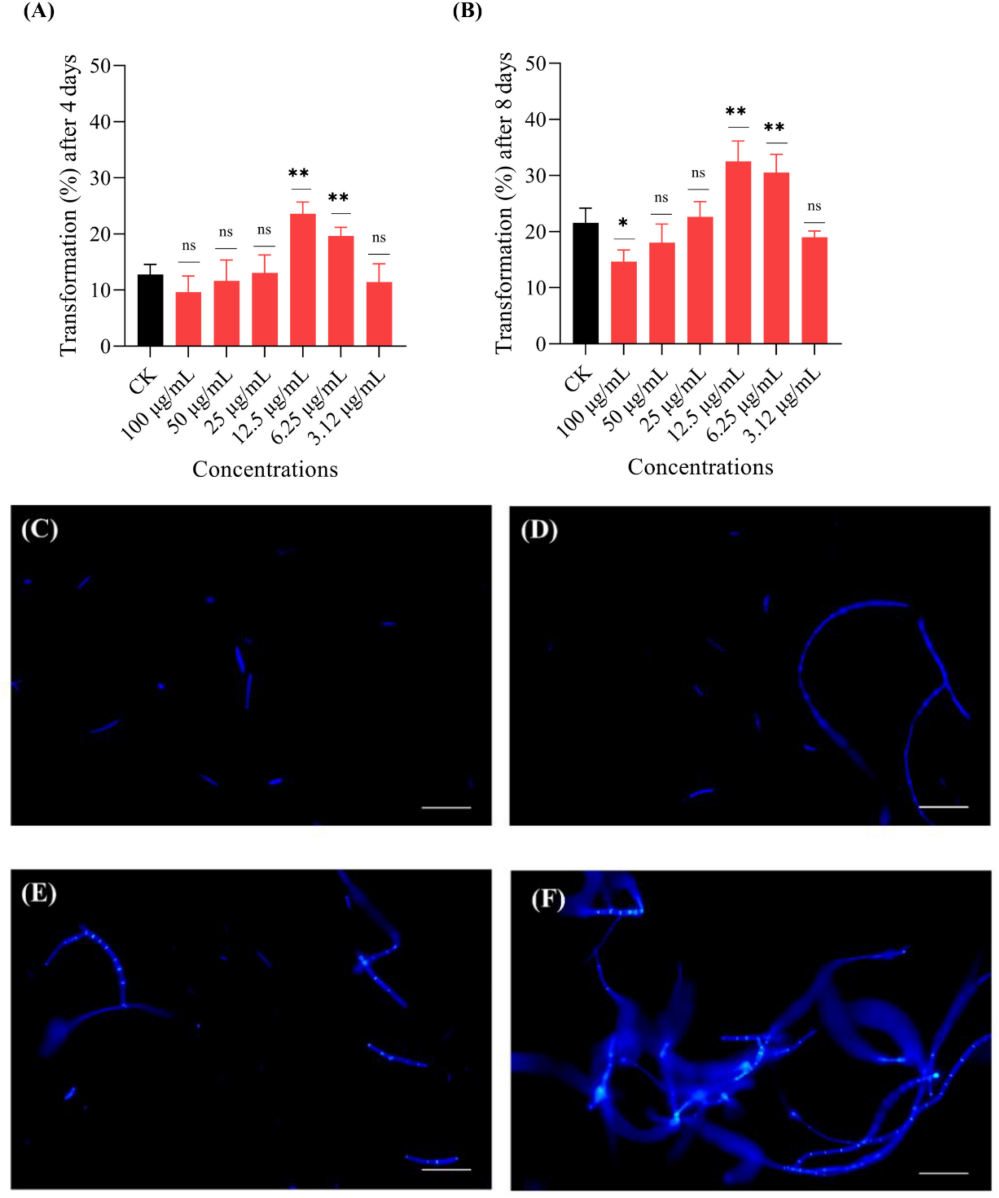
Effect of tryptophan on *Ophiocordyceps sinensis* blastospore-to-hyphae transformation. **(A,B)** Transformation (%) after treatment with tryptophan at different concentrations on day 4 **(A)** and day 8 **(B)**. **(C,D)** Confocal microscopic images of the control group (CK) at day 4 **(C)** and day 8 **(D)**. **(E,F)** Confocal microscopic images of tryptophan-treated group (12.5 μg/mL) at day 4 **(E)** and day 8 **(F)**. Scale bar = 50 μm. Data are presented as mean ± SD and analyzed using one-way ANOVA followed by Dunnett’s *post-hoc* test (*p < 0.05, **p < 0.01, ns, not significant).

Our results indicated that tryptophan treatment significantly enhanced blastospore-to-hyphae transformation in a concentration-dependent manner. At 12.5 μg/mL, transformation increased from 12.7% in the control to 23.6% on day 4, and from 21.5 to 31.5% on day 8 (Figure 9). Higher concentrations (100 μg/mL) significantly reduced transformation to 14.6%. Similarly, phenylalanine at 50 and 100 μg/mL decreased transformation on day 8 (16.2 and 14.6%, respectively), whereas cyclo(L-Pro-L-Val) and cyclo(L-Pro-L-Leu) had no significant effect at any tested concentration (Supplementary Figure S5). These results suggest that tryptophan functions as a key signaling metabolite promoting *O. sinensis* differentiation.

### 3.7 Bacterial supernatant is a rich source of extracellular tryptophan

**FIGURE 10:**
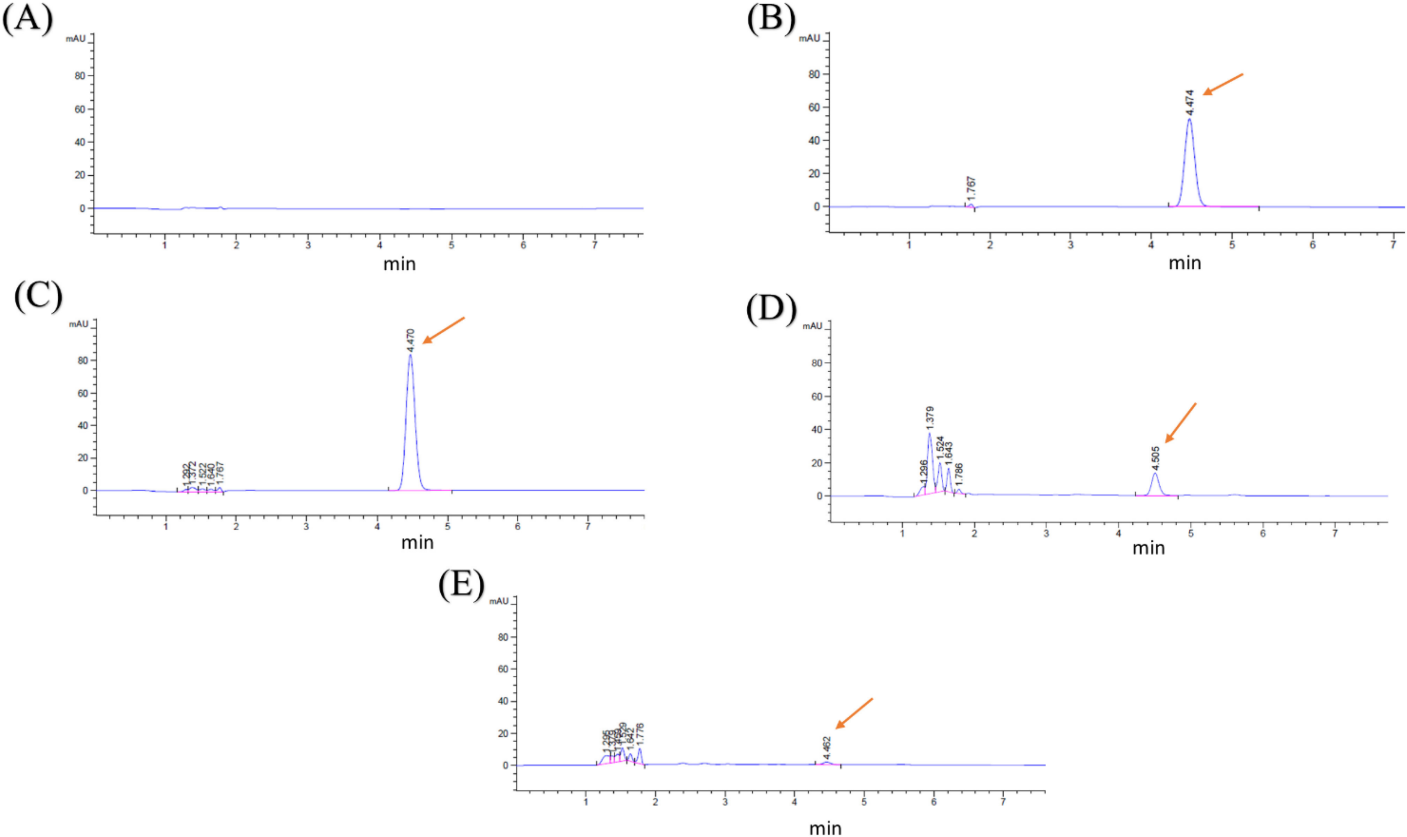
Quantification of tryptophan in microbial culture supernatants. Representative HPLC chromatograms of tryptophan detection: **(A)** blank control, **(B)** tryptophan standard (100 μg/mL), **(C)** *Bacillus amyloliquefaciens* supernatant containing 156.99 μg/mL tryptophan, **(D)** un-inoculated culture medium containing 21.0 μg/mL tryptophan, and **(E)** *Ophiocordyceps sinensis* supernatant containing only trace amounts of tryptophan (2.96 μg/mL).

To further confirm the role and to establish the origin of the tryptophan signal promoting fungal transformation, we quantified the extracellular tryptophan in the culture supernatants of both *O. sinensis* and *B. amyloliquefaciens*. Representative HPLC chromatograms of the blank control and tryptophan standard are shown in Figures 10A,B, respectively.

HPLC analysis confirmed a stark contrast in tryptophan concentration between the two sources. The culture supernatant of *B. amyloliquefaciens* contained 156.99 μg/mL of tryptophan (Figure 10C). It is worth mentioning here that the un-inoculated culture medium contained 21.0 μg/mL (Figure 10D) under identical conditions, representing a net increase of 136.99 μg/mL. Thus, bacterial activity contributed approximately 87% of the total tryptophan detected in the fermentation broth. This net increase reflects active bacterial tryptophan biosynthesis and secretion exceeding any consumption during growth. However, there are other metabolites in the supernatant, and future studies using non-targeted metabolomics will help determine its precise proportional abundance relative to other bacterial metabolites. When used in our bioassay at 5% (v/v), this yields an effective concentration of 7.8 μg/mL, which approaches the active range of pure tryptophan (6–12 μg/mL; section 3.6). Notably, despite this slightly lower tryptophan concentration, the bacterial supernatant induced substantially greater transformation (58.8%) than pure tryptophan at its optimal concentration (31.5%), suggesting that additional bacterial metabolites act synergistically with tryptophan to enhance morphogenesis. In contrast, the supernatant from *O. sinensis* culture contained only trace amounts of tryptophan (2.96 μg/mL) (Figure 10E).

These results clearly establishes the bacterium, and not the fungus, as the primary source of the extracellular tryptophan signal. While *O. sinensis* is capable of producing basal levels of tryptophan necessary for its primary metabolism, the quantity is insufficient to efficiently drive the morphogenetic program. The abundant secretion of tryptophan by *B. amyloliquefaciens* provides a supra-physiological, cross-kingdom signal that modulates the fungal TOR-*BRG1* axis, thereby overcoming the intrinsic metabolic limitation and significantly increasing the blastospore-to-hypha transformation rate. This demonstrates a synergistic interaction where a bacterial symbiont supplies a key metabolic signal to potentiate the developmental transition of *O. sinensis.*

### 3.8 Tryptophan modulate TOR–*BRG1* signaling to promote *O. sinensis* transformation

**FIGURE 11:**
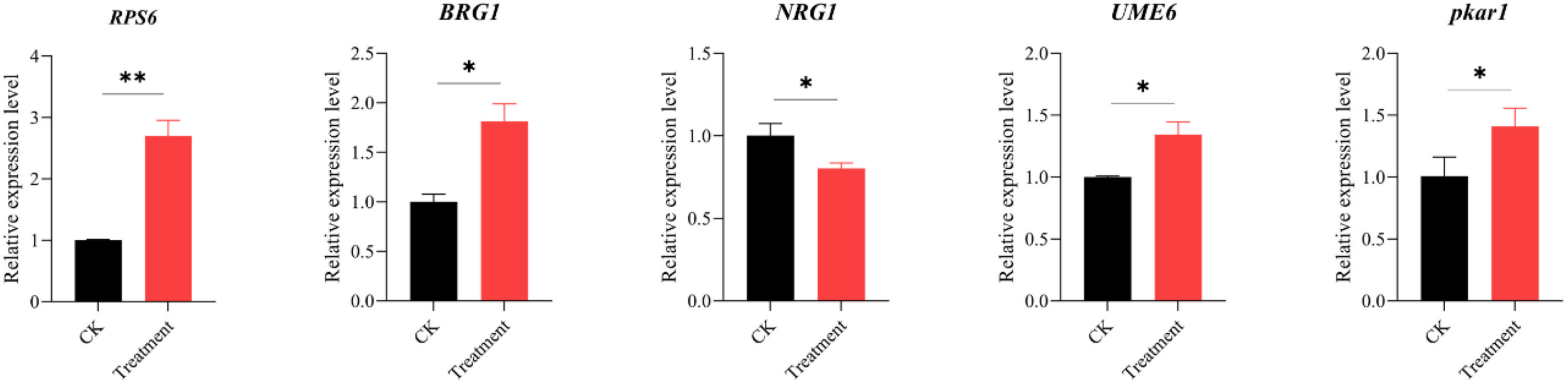
Effect of tryptophan on the expression of TOR pathway and hyphae-associated genes in *Ophiocordyceps sinensis* at day 4. Data are presented as mean ± SD and analyzed using a two-tailed unpaired Student’s *t*-test (*p < 0.05, **p < 0.01, ns, not significant).

Our results with qRT-PCR analysis showed that tryptophan treatment (12.5 μg/mL) significantly increased the expression of TOR pathway and hyphae-associated genes in *O. sinensis* at day 4 (Figure 11). *RPS6* was upregulated, indicating enhanced TOR signaling. *BRG1*, *UME6*, and *pkar1* were also significantly upregulated, while *NRG1* expression decreased compared to the control. These results indicate that tryptophan promotes blastospore-to-hyphae transformation by activating the TOR–*BRG1* signaling network.

### 3.9 Tryptophan enhances hyphal development by upregulating cell wall remodeling enzymes in *O. sinensis*

**FIGURE 12:**
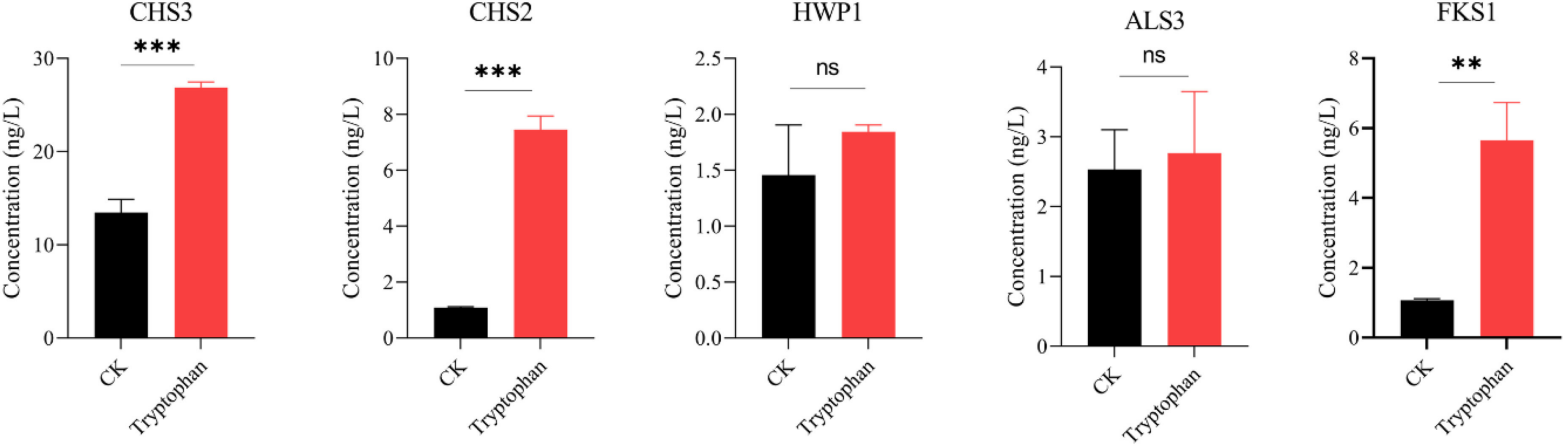
Quantification of cell wall remodeling enzymes in *Ophiocordyceps sinensis* following tryptophan treatment (12.5 μg/mL). Data are presented as mean ± SD and analyzed using a two-tailed unpaired Student’s *t*-test (**p < 0.01, ***p < 0.001, ns, not significant).

To confirm that the transcriptional upregulation translated to increased synthesis of functional enzymes, we quantified the abundance of key cell wall biosynthesis enzymes. Our results showed that CHS2, CHS3, and FKS1 were significantly upregulated on day 4 after tryptophan treatment (12.5 μg/mL). Specifically, CHS2 increased from 1.08 ng/L in the control to 7.45 ng/L in the treated group, while CHS3 increased from 13.45 to 26.89 ng/L, and FKS1 increased from 1.06 to 5.64 ng/L. In contrast, HWP1 and ALS3 levels were not significantly affected. These results indicate that tryptophan promotes blastospore-to-hyphae transformation by specifically enhancing the expression of key cell wall biosynthesis enzymes (CHS2, CHS3, and FKS1) (Figure 12).

## 4 Discussion

*Ophiocordyceps sinensis* is a unique entomopathogenic fungus that parasitizes the larvae of *Thitarodes* spp., eventually forming the highly valued Chinese cordyceps. Its complex life cycle involves the transformation of fungal blastospores into hyphae within the insect host, a process that is crucial for successful infection and host mummification. However, this transformation is slow and often inefficient under artificial conditions, limiting large-scale cultivation (Chen et al., 2013). In nature, *O. sinensis* exists in a complex ecological niche, and mounting evidence suggests that associated microorganisms especially bacteria can play a supportive or even regulatory role in its development and differentiation (Liang et al., 2019; Wu et al., 2020). Insect microbiota have been implicated in providing nutritional factors, signaling molecules, or secondary metabolites that influence fungal growth and host–fungus interactions (Alam et al., 2021; Pawlowska, 2024).

In this study, we demonstrated that the culture supernatant of *Bacillus amyloliquefaciens*, an associated bacterial isolate from *Thitarodes* larvae, markedly enhanced the blastospore-to-hyphae transformation of *O. sinensis* suggesting that diffusible bacterial metabolites play a key role in stimulating fungal differentiation. Similar facilitative interactions between *Bacillus* species and fungi have been reported in other systems, where bacterial metabolites modulate fungal morphogenesis, cell wall remodeling, or secondary metabolism (Andrić et al., 2020; Calvo et al., 2002; Zhang et al., 2025). The strong promotive effect of the supernatant observed here indicates that *B. amyloliquefaciens* produces cross-kingdom signaling molecules capable of activating intrinsic developmental pathways in *O. sinensis*.

Transcriptomic analysis revealed that *B. amyloliquefaciens* supernatant triggered early transcriptional reprogramming in *O. sinensis*, with activation of amino acid, carbohydrate, and energy metabolism pathways supporting morphogenesis. Especially, the significant upregulation of the transcription factor *BRG1* and the TOR effector *RPS6* suggests that bacterial metabolites modulate the TOR–*BRG1* signaling axis, which coordinates nutrient sensing with developmental progression. The TOR–*BRG1* association observed here resembles regulatory mechanisms described in other fungi, where TOR signaling modulates chromatin remodeling and the expression of hypha-specific genes (Dong et al., 2025; Li et al., 2025; Su et al., 2013; Vangalis et al., 2023). We utilized rapamycin to specifically target the mechanistic target of rapamycin (mTOR) kinase that integrates nutrient and growth signals (Lamming, 2016). Functional validation using rapamycin confirmed that TOR signaling controls transformation in a concentration-dependent manner. Our results indicated moderate reduction of TOR promotes the *BRG1*-associated developmental network, while excessive inhibition shifts the system toward repression, highlighting the delicate balance of TOR activity required for morphogenesis. This delicate balance of TOR activity is consistent with mechanisms in the opportunistic pathogen *Candida albicans*, where a specific, sub-inhibitory reduction in Tor1 signaling is required to activate the transcription factor *BRG1*. *BRG1* then orchestrates a developmental switch by both recruiting chromatin remodelers to displace the hyphal repressor *NRG1* and activating the key downstream target *UME6*, driving sustained morphogenesis (Lu et al., 2012).

The butanol fraction of *B. amyloliquefaciens* extract significantly enhanced transformation, while the ethyl acetate fraction showed no effect, indicating that polar metabolites mediate the activity. Four compounds were identified—phenylalanine, tryptophan, cyclo(L-Pro-L-Val), and cyclo(L-Pro-L-Leu)—with tryptophan showing the strongest, concentration-dependent enhancement of spore-to-hypha transformation. qRT-PCR analysis revealed that tryptophan treatment upregulated *RPS6*, *BRG1*, *UME6*, and *pkar1*, and downregulated *NRG1*, confirming activation of the TOR–*BRG1* signaling network. It is important to distinguish between the nutritional and signaling functions of tryptophan. At the optimal concentration used in this study (12.5 μg/mL), tryptophan is present at sub-nutritional levels that are insufficient to support significant biomass accumulation but are sufficient to activate TOR signaling. This is consistent with the emerging paradigm that amino acids and other natural small molecules can function as bona fide signaling molecules at concentrations well below their metabolic requirements. The biphasic dose-response curve further supports a signaling role, as true nutrients typically show monotonic, not hormetic, responses. Similar signaling roles for amino acids and natural metabolites have been described in other systems (Wang et al., 2025). Furthermore, high concentrations of tryptophan may inhibit transformation by affecting the TOR pathway, leading to feedback inhibition or metabolic imbalance, as imbalance amino acid signaling can dysregulate nutrient sensing and repress morphogenesis. This is consistent with our rapamycin experiments (Figure 5), where excessive TOR inhibition (50 μg/mL rapamycin) suppressed transformation and upregulated the repressor *NRG1* while downregulating *BRG1* and *UME6* (Figure 6B). These results suggest that tryptophan functions as a signaling molecule that promotes *O. sinensis* transformation, consistent with its recognized role as an inter-kingdom modulator of microbial and host physiology (Grifka-Walk et al., 2021; Jamshed et al., 2022). Tryptophan biosynthesis is crucial for microbial growth (Martins et al., 2024) and is a key regulator of the host immune homeostasis (Pei et al., 2025). While our data identify tryptophan as a key signal, other metabolites in the *B. amyloliquefaciens* supernatant may act synergistically with tryptophan to modulate the response in a more robust manner. Furthermore, non-targeted metabolomics of the *B. amyloliquefaciens* supernatant could reveal additional synergistic metabolites. This represents an exciting avenue for future research.

Collectively, these results support a model in which bacterial metabolites, particularly tryptophan, serve as a signaling molecule that modulate TOR signaling to a level permissive for downstream *BRG1*-dependent transcription, leading to enhanced transformation of *O. sinensis*. This proposed sequence of events within the TOR-*BRG1* signaling axis is summarized in the Graphical abstract. Understanding this cross-kingdom signaling network not only provides insight into the natural symbiosis between *B. amyloliquefaciens* and *O. sinensis* but also offers a promising strategy for improving artificial cultivation by supplementing specific bacterial metabolites that trigger fungal differentiation.

## 5 Conclusion

*Bacillus amyloliquefaciens* promotes the blastospore-to-hyphae transformation of *Ophiocordyceps sinensis* through the production of diffusible metabolites, particularly tryptophan. These metabolites modulate the TOR–*BRG1* signaling axis, leading to transcriptional reprogramming that supports *O. sinensis* morphogenesis. This study provides a potential strategy to enhance artificial cultivation of *O. sinensis*.

## Data Availability

The datasets presented in this study can be found in online repositories. The names of the repository/repositories and accession number(s) can be found in the article/Supplementary material.

## References

[B1] Ahsan T. Zang C. Yu S. Pei X. Xie J. Lin Y. et al. (2022). Screening, and optimization of fermentation medium to produce secondary metabolites from Bacillus amyloliquefaciens, for the biocontrol of early leaf spot disease, and growth promoting effects on peanut (Arachis hypogaea L.). *J. Fungi* 8:1223. 10.3390/jof8111223 36422044 PMC9698727

[B2] Alam B. Lǐ J. Gě Q. Khan M. A. Gōng J. Mehmood S. et al. (2021). Endophytic fungi: From symbiosis to secondary metabolite communications or vice versa? *Front. Plant Sci.* 12:791033. 10.3389/fpls.2021.791033 34975976 PMC8718612

[B3] Andrić S. Meyer T. Ongena M. (2020). Bacillus responses to plant-associated fungal and bacterial communities. *Front. Microbiol.* 11:1350. 10.3389/fmicb.2020.01350 32655531 PMC7324712

[B4] Calvo A. Wilson R. Bok J. Keller N. (2002). Relationship between secondary metabolism and fungal development. *Microbiol. Mol. Biol. Rev.* 66 447–459. 10.1128/MMBR.66.3.447-459.2002 12208999 PMC120793

[B5] Chai W. Mao X. Li C. Zhu L. He Z. Wang B. (2024). Neurotransmitter acetylcholine mediates the mummification of Ophiocordyceps sinensis-infected Thitarodes xiaojinensis larvae. *Appl. Environ. Microbiol.* 90:e0033324. 10.1128/aem.00333-24 39109874 PMC11409639

[B6] Chen P. Wang S. Nie S. Marcone M. (2013). Properties of cordyceps Sinensis: A review. *J. Funct. Foods* 5 550–569. 10.1016/j.jff.2013.01.034 32288794 PMC7104941

[B7] Chou T. Kuo H. Tsai S. Huang S. Yang M. Lee S. et al. (2021). Doubled production of cordycepin analogs in cultured Cordyceps militaris by addition of Andrea droppings. *Nat. Prod. Res.* 35 5459–5464. 10.1080/14786419.2020.1781112 32594773

[B8] Dai Y. Wu C. Yuan F. Wang F. Huang L. Chen Z. et al. (2020). Evolutionary biogeography on *ophiocordyceps sinensis*: An indicator of molecular phylogeny to geochronological and ecological exchanges. *Geosci. Front.* 11 807–820. 10.1016/j.gsf.2019.09.001

[B9] Dong Y. Aflaki F. Mozgova I. Berr A. (2025). TORquing chromatin: The regulatory role of TOR kinase in chromatin function. *J. Exp. Bot.* 76 2405–2418. 10.1093/jxb/erae474 39565832

[B10] Grifka-Walk H. Jenkins B. Kominsky D. (2021). Amino acid trp: The far out impacts of host and commensal tryptophan metabolism. *Front. Immunol.* 12:653208. 10.3389/fimmu.2021.653208 34149693 PMC8213022

[B11] Han R. Wu H. Tao H. (2019). *Research on Chinese Cordyceps During the Past 70 Years in China.* Beijing: Editorial Department of the Chinese Journal of Applied Entomology, 849–883.

[B12] Jamshed L. Debnath A. Jamshed S. Wish J. Raine J. Tomy G. et al. (2022). An emerging cross-species marker for organismal health: Tryptophan-kynurenine pathway. *Int. J. Mol. Sci.* 23:6300. 10.3390/ijms23116300 35682980 PMC9181223

[B13] Khalid M. Zheng X. Han R. Cao L. (2025). Fusarium pseudonygamai promotes blastospore transformation in Ophiocordyceps sinensis: Insights into microbial interaction and key mechanisms. *J. Fungi* 11:746. 10.3390/jof11100746 41149935 PMC12565527

[B14] Kim M. Cravener M. Solis N. Filler S. Mitchell A. P. (2024). A Brg1-Rme1 circuit in Candida albicans hyphal gene regulation. *mBio* 15:e0187224. 10.1128/mbio.01872-24 39078139 PMC11389389

[B15] Lamming D. (2016). Inhibition of the mechanistic target of rapamycin (mTOR)-rapamycin and beyond. *Cold Spring Harb. Perspect. Med.* 6:a025924. 10.1101/cshperspect.a025924 27048303 PMC4852795

[B16] Lee D. Lee S. Kang K. Kim K. (2021). Bioactive phytochemicals from mulberry: Potential anti-inflammatory effects in lipopolysaccharide-stimulated RAW 264.7 macrophages. *Int. J. Mol. Sci.* 22:8120. 10.3390/ijms22158120 34360887 PMC8348635

[B17] Li Y. Sun S. Li G. Yang Z. Xing Y. Wang R. et al. (2025). The TOR signaling pathway governs fungal development, virulence and ustiloxin biosynthesis in ustilaginoidea virens. *J. Fungi* 11:239. 10.3390/jof11040239 40278060 PMC12028740

[B18] Liang Y. Hong Y. Mai Z. Zhu Q. Guo L. (2019). Internal and external microbial community of the thitarodes moth, the host of Ophiocordyceps sinensis. *Microorganisms* 7:517. 10.3390/microorganisms7110517 31683719 PMC6920881

[B19] Liu G. Cao L. Qiu X. Han R. (2020). Quorum Sensing activity and hyphal growth by external stimuli in the Entomopathogenic fungus Ophiocordyceps sinensis. *Insects* 11:205. 10.3390/insects11040205 32225083 PMC7240566

[B20] Liu G. Han R. Cao L. (2019). Artificial cultivation of the Chinese cordyceps from injected ghost moth larvae. *Environ. Entomol.* 48 1088–1094. 10.1093/ee/nvz099 31517384

[B21] Liu G. Zheng X. Long H. Rao Z. Cao L. Han R. (2021). Gut bacterial and fungal communities of the wild and laboratory-reared thitarodes larvae, host of the Chinese Medicinal fungus Ophiocordyceps sinensis on Tibetan Plateau. *Insects* 12:327. 10.3390/insects12040327 33916889 PMC8067570

[B22] Lo H. Hsieh C. Lin F. Hsu T. H. (2013). A systematic review of the mysterious caterpillar fungus Ophiocordyceps sinensis in Dong-ChongXiaCao (Dōng Chóng Xià Cǎo) and related bioactive ingredients. *J. Tradit. Complement. Med.* 3 16–32. 10.4103/2225-4110.106538 24716152 PMC3924981

[B23] Lu Y. Su C. Liu H. (2012). A GATA transcription factor recruits Hda1 in response to reduced Tor1 signaling to establish a hyphal chromatin state in Candida albicans. *PLoS Pathog.* 8:e1002663. 10.1371/journal.ppat.1002663 22536157 PMC3334898

[B24] Martins N. Viana M. Maigret B. (2024). Fungi tryptophan synthases: What is the role of the linker connecting the α and β structural domains in Hemileia vastatrix TRPS? *A Molecular Dynamics Investigation. Molecules* 29:756. 10.3390/molecules29040756 38398508 PMC10893352

[B25] Meng Q. Yu H. Zhang H. Zhu W. Wang M. Zhang J. et al. (2015). Transcriptomic insight into the immune defenses in the ghost moth, Hepialus xiaojinensis, during an Ophiocordyceps sinensis fungal infection. *Insect Biochem Mol Biol.* 64 1–15. 10.1016/j.ibmb.2015.06.014 26165779

[B26] Pawlowska T. (2024). Symbioses between fungi and bacteria: From mechanisms to impacts on biodiversity. *Curr. Opin. Microbiol.* 80:102496. 10.1016/j.mib.2024.102496 38875733 PMC11323152

[B27] Pei T. Li W. Zhou Z. Zhang Q. Yu G. Yin S. et al. (2025). The relationship between tryptophan metabolism and gut microbiota: Interaction mechanism and potential effects in infection treatment. *Microbiol. Res.* 298:128211. 10.1016/j.micres.2025.128211 40393170

[B28] Qin Q. Zhou G. Zhang H. Meng Q. Zhang J. Wang H. et al. (2018). Obstacles and approaches in artificial cultivation of Chinese cordyceps. *Mycology* 9 7–9. 10.1080/21501203.2018.1442132 30123655 PMC6059063

[B29] Qu H. Guo Z. Ma L. Zhang X. Ma H. Chen Y. (2022). Antifungal effects and active compounds of the leaf of Allium mongolicum Regel. *Front. Chem.* 10:993893. 10.3389/fchem.2022.993893 36092670 PMC9451007

[B30] Rao Z. Cao L. Wu H. Qiu X. Liu G. Han R. (2019). Comparative transcriptome analysis of thitarodes armoricanus in response to the entomopathogenic fungi paecilomyces hepiali and Ophiocordyceps sinensis. *Insects* 11:4. 10.3390/insects11010004 31861642 PMC7022891

[B31] Sharma J. Rosiana S. Razzaq I. Shapiro R. (2019). Linking cellular morphogenesis with antifungal treatment and susceptibility in Candida pathogens. *J. Fungi* 5:17. 10.3390/jof5010017 30795580 PMC6463059

[B32] Su C. Lu Y. Liu H. (2013). Reduced TOR signaling sustains hyphal development in Candida albicans by lowering Hog1 basal activity. *Mol. Biol. Cell.* 24 385–397. 10.1091/mbc.E12-06-0477 23171549 PMC3564525

[B33] Sun T. Zou W. Luo R. Li C. Zhang C. Yu H. (2023). Compositional and functional diversities of core microbial communities in wild and artificial Ophiocordyceps sinensis. *Int. Microbiol.* 26 791–806. 10.1007/s10123-023-00333-5 36781511

[B34] Vangalis V. Markakis E. Knop M. Pietro A. Typas M. Papaioannou I. (2023). Components of TOR and MAP kinase signaling control chemotropism and pathogenicity in the fungal pathogen Verticillium dahliae. *Microbiol. Res.* 271:127361. 10.1016/j.micres.2023.127361 36921400

[B35] Wang Y. Peng W. Li C. Wu Y. Sheng Y. Zi C. et al. (2025). The improvement effect of ellagic acid and urolithins on metabolic diseases: Pharmacology and mechanism. *Food Med. Homol.* 2:9420058. 10.26599/FMH.2025.9420058

[B36] Wei Y. Zhang L. Wang J. Wang W. Niyati N. Guo Y. et al. (2021). Chinese caterpillar fungus (Ophiocordyceps sinensis) in China: Current distribution, trading, and futures under climate change and overexploitation. *Sci. Total Environ.* 755:142548. 10.1016/j.scitotenv.2020.142548 33035977 PMC7521209

[B37] Wu H. Cao L. He M. Han R. De Clercq P. (2021). Interspecific hybridization and complete mitochondrial genome analysis of two ghost moth species. *Insects* 12:1046. 10.3390/insects12111046 34821846 PMC8625261

[B38] Wu H. Rao Z. Cao L. De Clercq P. Han R. (2020). Infection of Ophiocordyceps sinensis fungus causes dramatic changes in the microbiota of its thitarodes host. *Front. Microbiol.* 11:577268. 10.3389/fmicb.2020.577268 33343519 PMC7744566

[B39] Wu P. Qin Q. Zhang J. Zhang H. Li X. Wang H. et al. (2022). The invasion process of the entomopathogenic fungus Ophiocordyceps sinensis into the larvae of ghost moths (Thitarodes xiaojinensis) using a GFP-labeled strain. *Front. Microbiol.* 13:974323. 10.3389/fmicb.2022.974323 36118238 PMC9479185

[B40] Yoshinari T. Akiyama T. Nakamura K. Kondo T. Takahashi Y. Muraoka Y. et al. (2007). Dioctatin A is a strong inhibitor of aflatoxin production by Aspergillus parasiticus. *Microbiology* 153 2774–2480. 10.1099/mic.0.2006/005629-0 17660441

[B41] Zeng Z. Mou D. Luo L. Zhong W. Duan L. Zou X. (2021). Different cultivation environments affect the yield, bacterial community and metabolites of Cordyceps cicadae. *Front. Microbiol.* 12:669785. 10.3389/fmicb.2021.669785 34046024 PMC8144455

[B42] Zhang M. Zhang Y. Zhao Z. Deng F. Jiang H. Liu C. et al. (2025). Bacterial-fungal interactions: Mutualism, antagonism, and competition. *Life* 15:1242. 10.3390/life15081242 40868890 PMC12387163

