# Supplementary material for: A cross-kingdom metabolite signal: tryptophan from Bacillus amyloliquefaciens triggers Ophiocordyceps sinensis morphogenesis via the TOR-BRG1 pathway
Source: Front Microbiol. 2026 Jun 1;17:1818098. doi: 10.3389/fmicb.2026.1818098 (PMC13267341; doi:10.3389/fmicb.2026.1818098)
Supplement: Supplementary file 1 [file Data_sheet_1.pdf]

Supplementary Material

1 Supplementary Figures and Tables

Table S1. Supplementary Table S1: Primer's sequence

Name	Primer Sequences (*5'-3'*)
IDH1	CGACCACGCCAACCGCATCT
	CGAGAATGGCCCGCGTAAACT
ilv-2	AAAACGGCAAGTCGTGGAAGG
	AGCGTCGCTGAGGAGGTTCA
CDase	CAACCTCACCGTCGGCAACA
	GGATGAAGGAAAGCGCCCTATT
eca39	GGAGGCTGGTCTCGGGAATG
	GCTAGGGCTGACAATGATGAGGA
stt3	TACCGCGAGGCATATCAGTG
	TGTGCGTGTTGTTCCAAGTGT
Dbt	GGGCAATGAGGGCGACAAGG
	CGGTGGCATGGCTCGAAGGT
PaAT-1	GAAAGATTCGGCGGAAGCAC
	CGACAATGACCAGGAACAGATACA
BEA3	GGTCCCTGATGTGGGTGGTA
	CCCTGCGAGTTGTCTTGGTC
CSY2	ACACCATTCCCATCACCAACA
	TCGGTAGAAGGGCAGGAGTTA
ARG1	GATACCTCGACCATCCTGAAATG
	GACCTCGCCAAAGTCTTCCTC
RPS6	CATCAGCTACCCTGCCAACGG
	CCTGCTTCATCGGGAAACCCT
BRG1	AGGAGGAGCTTGATGCCGTGTC
	CGCCCGAGGATAATGAAGAGGA
NRG1	CGTCAATCGACTACGCCTACCC
	ACGTGGCCCTGCTGCTATCC
UME6	CGGCGCAACAAGCTCGACTG
	GGACACTGCGACGACTACCCAAGA
pkar1	GTGACGCAGGCGACTTCTTT
	TCAATGGTGCCGACCTTGTT
TUB1	CGCTCCGTCTCCATGCTTTC
	CGACTTCCTCGTAGTCCTTCTCG
TEF3	TTCTCCTGAACCAGACCCACC
	CGACTTGCTCGTTGTTGATGG
act	TCCGAGACATCAAGGAGAAGC
	TCAAGACCGAGGACAGAGGG
TUBB	GCGTCGTCCTGATTGTCGTTC
	CATTTCCTTTCTGCGCTTTCG

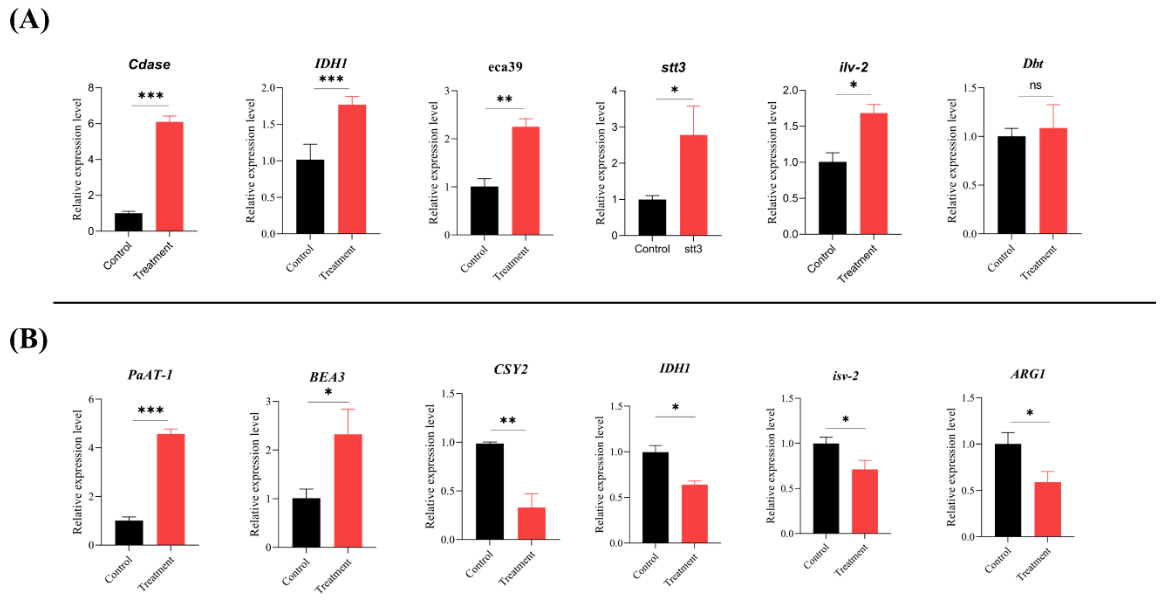

Figure S1. qRT-PCR validation of RNA-seq data for selected differentially expressed genes. **(A)** Relative mRNA expression of genes *IDH1*, *ilv-2*, *Cdase*, *eca39*, *stt3*, and *Dbt* in *Ophiocordyceps sinensis* after 4 days of treatment with *B. amyloliquefaciens* supernatant. **(B)** Relative mRNA expression of genes *PaAT-1*, *BEA3*, *CSY2*, *ilv-2*, *ARG1*, and *IDH1* after 8 days of treatment.

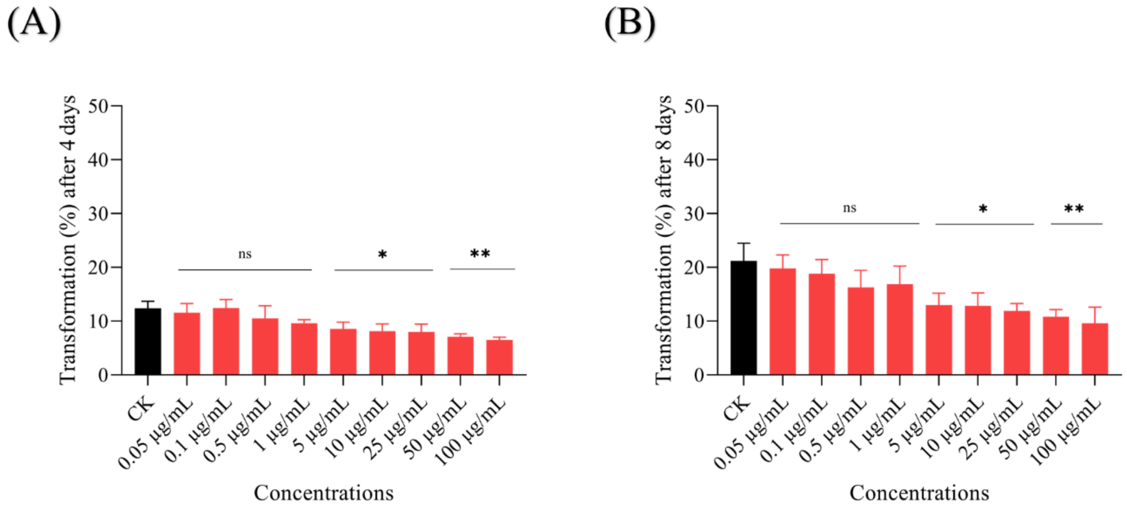

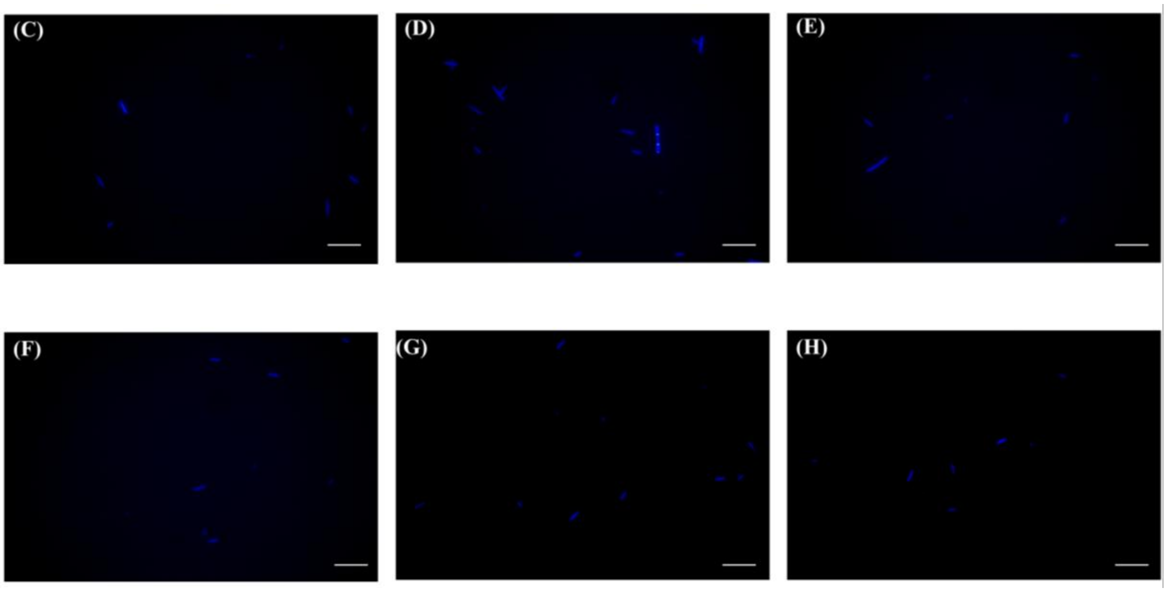

Figure S2. The TOR inhibitor farnesol suppresses *Ophiocordyceps sinensis* blastospore-to-hypha transformation. Transformation percentage of *O. sinensis* blastospores following treatment with a range of farnesol concentrations (0.05 – 100 µg/mL) after (**A**) 4 days and (**B**) 8 days of incubation. **(C,D)** Confocal microscopic images of blastospores from control grpup (CK) at day 4 and day 8. (**E,F**) Confocal microscopic images of blastospores treated with higher concentrations of farnesol (50 µg/mL) at day 4 and day 8. (**G,H**) Images of spores treated with lower concentrations of farnesol (0.5 µg/mL) at day 4 and day 8. Scale bar = 50 µm. Data are presented as mean ± SD and analyzed using one-way ANOVA followed by Dunnett's post hoc test (**p* < 0.05, ***p* < 0.01, ns = not significant).

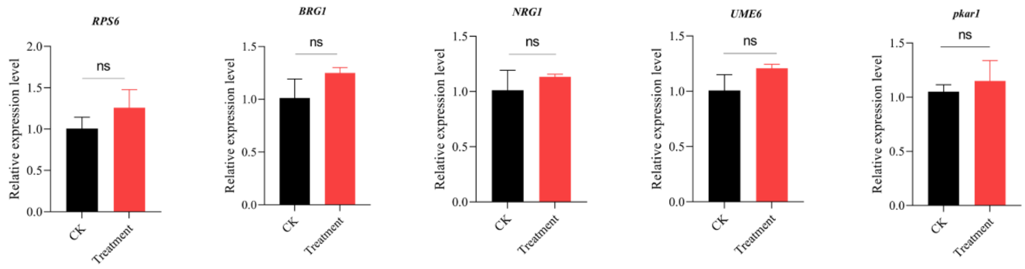

Figure S3. Farnesol treatment does not alter the expression of the TOR-*BRG1* regulatory network. Relative mRNA expression levels of *RPS6, BRG1, UME6, NRG1*, and *pkar1* in *Ophiocordyceps sinensis* after treatment with a high concentration of farnesol (50 µg/mL) at day 4.

(A)

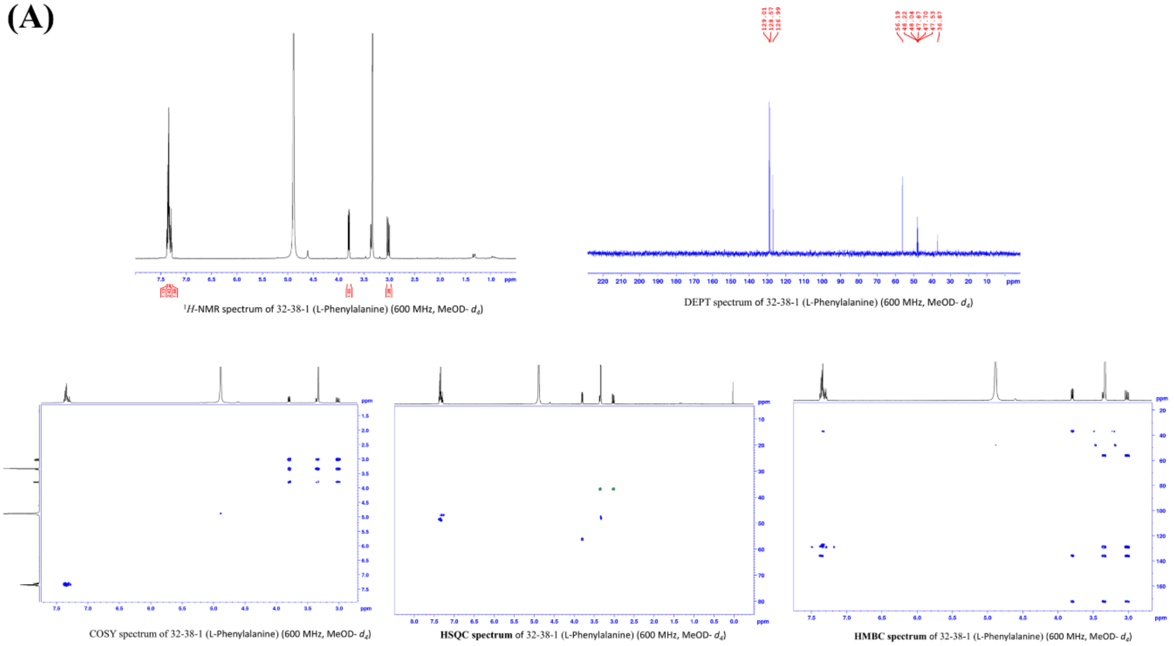

(B)

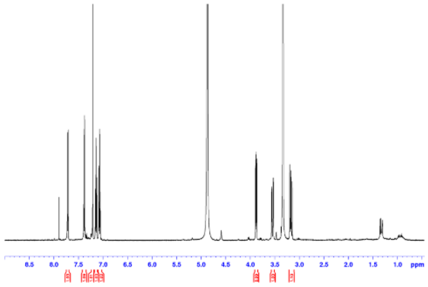

^{1}H-NMR spectrum of 32-38-2 (Tryptophan) (600 MHz, MeOD- d_4)

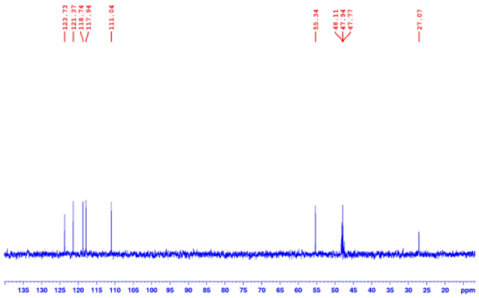

DEPT spectrum of 32-38-2 (Tryptophan) (600 MHz, MeOD- d_4)

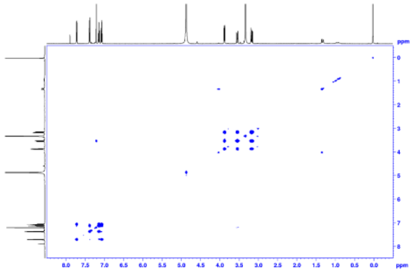

COSY spectrum of 32-38-2 (Tryptophan) (600 MHz, MeOD- d_4)

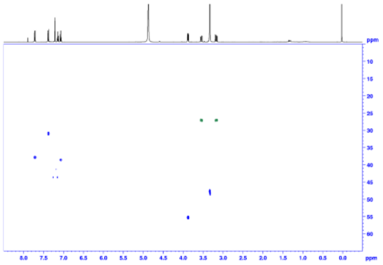

HSQC spectrum of 32-38-2 (Tryptophan) (600 MHz, MeOD- d_4)

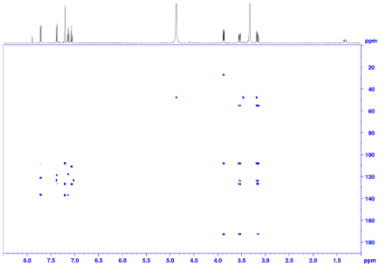

HMBC spectrum of 32-38-2 (Tryptophan) (600 MHz, MeOD- d_4)

(C)

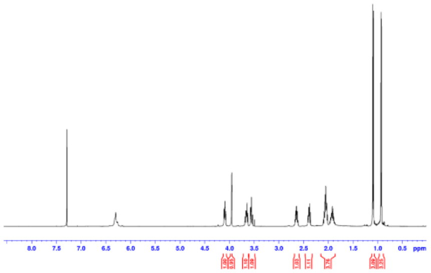

^{1}H-NMR spectrum of 35-38- (cyclo(L-Pro-L-Val)) (600 MHz, $CHCl_3$)

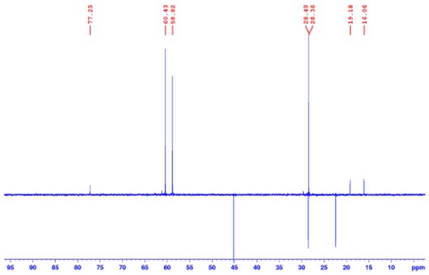

DEPT spectrum of 35-38- (cyclo(L-Pro-L-Val)) (600 MHz, $CHCl_3$)

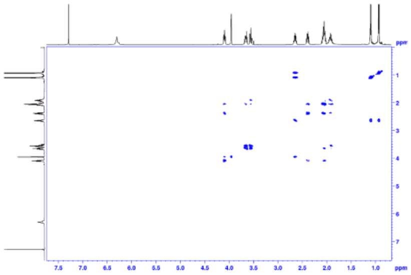

COSY spectrum of 35-38- (cyclo(L-Pro-L-Val)) (600 MHz, $CHCl_3$)

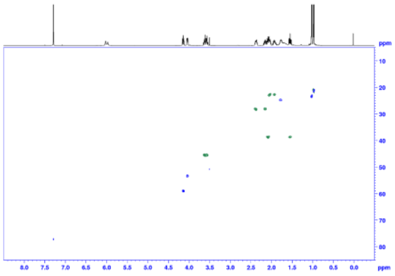

HSQC spectrum of 35-38- (cyclo(L-Pro-L-Val)) (600 MHz, $CHCl_3$)

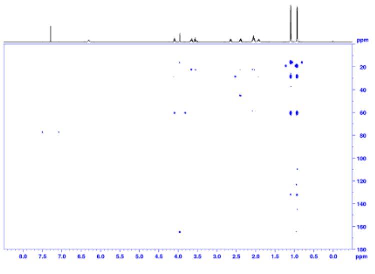

HMBC spectrum of 35-38- (cyclo(L-Pro-L-Val)) (600 MHz, $CHCl_3$)

(D)

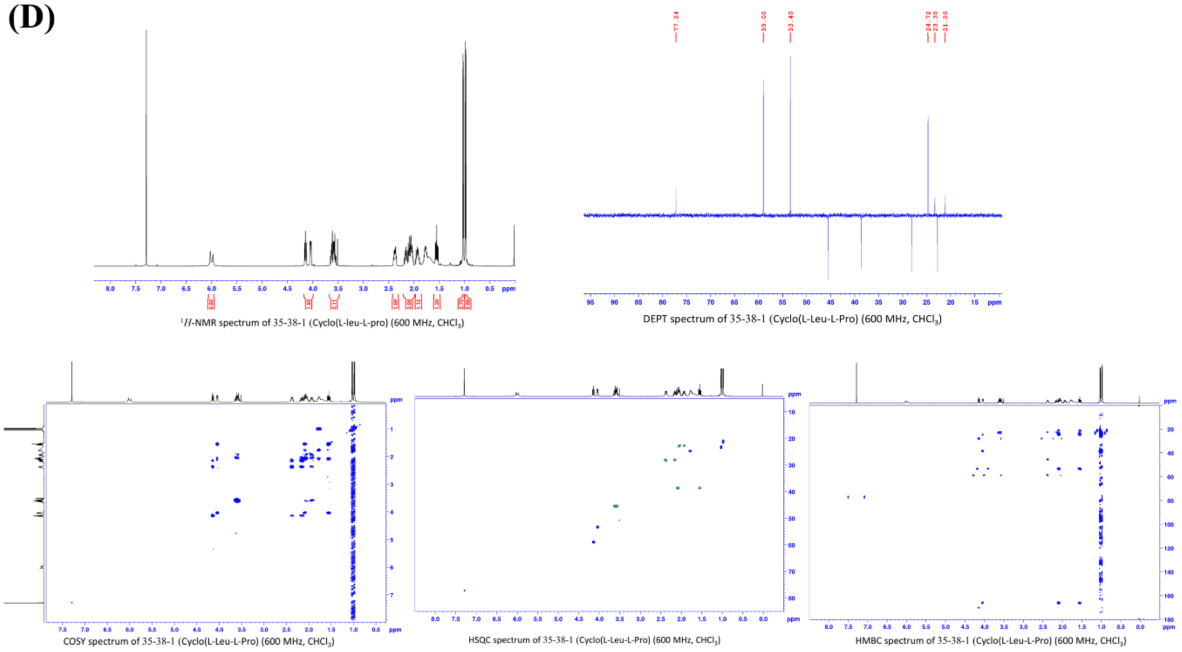

Figure S4. NMR spectra for structural elucidation of compounds isolated from the butanol crude extract of *Bacillus amyloliquefaciens*. Representative 1D and 2D NMR spectra used for the identification and structural confirmation of the four purified compounds: **(A)** L-phenylalanine, **(B)** Tryptophan, **(C)** Cyclo(L-Pro-L-Val), and **(D)** Cyclo(L-Leu-L-Pro). For each compound, ^{1}H, DEPT, COSY, HSQC, and HMBC spectra are shown.

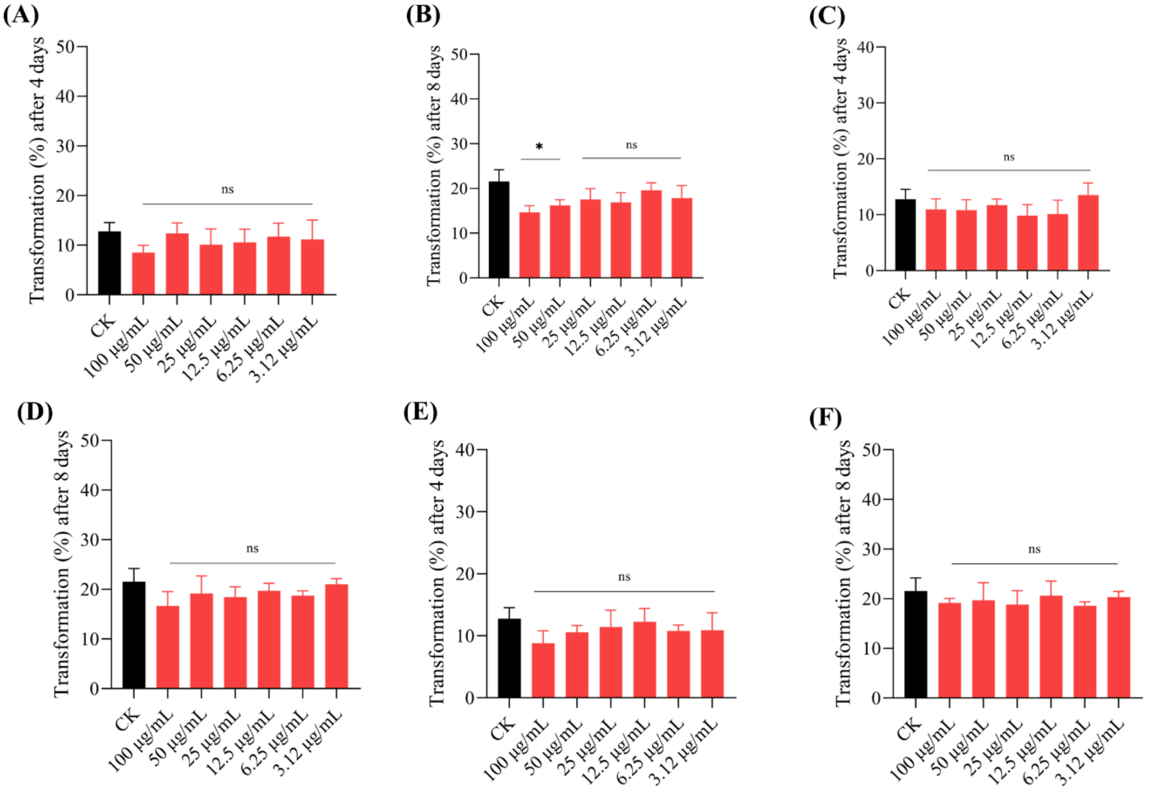

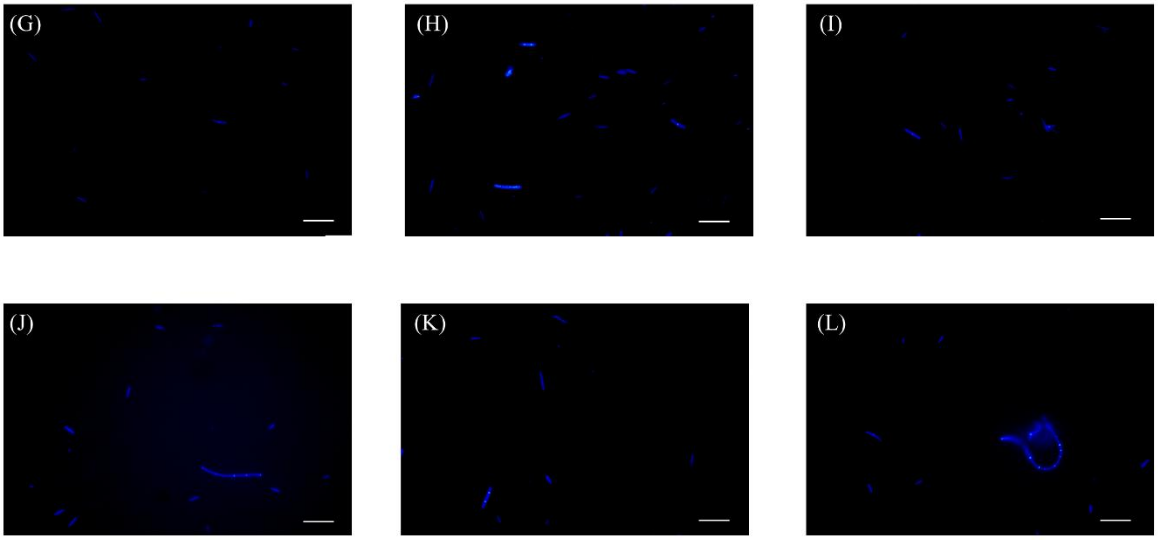

Figure S5. Isolated compounds other than tryptophan show minimal or inhibitory effects on *O. sinensis* transformation. Transformation percentage of *O. sinensis* blastospores after treatment with **(A,B)** L-phenylalanine, **(C,D)** cyclo(L-Pro-L-Val), and **(E-F)** cyclo(L-Leu-L-Pro) at various concentrations, measured on day 4 and day 8, respectively. **G,H**) Confocal microscopic images of blastospores from control grpup (CK) at day 4 and day 8. (**I,J**) Confocal microscopic images of L-phenylalanine-treated group (50 µg/mL) at day 4 (E) and day 8. (**K,L**) Confocal microscopic images of cyclo(L-Pro-L-Val)-treated group (12.5 µg/mL) at day 4 (E) and day 8. Scale bar = 50 µm. Data are presented as mean ± SD and analyzed using one-way ANOVA followed by Dunnett's post hoc test (*$p < 0.05$, ns = not significant).
